# Lipid profile of Mexican children with Down syndrome

**DOI:** 10.1186/s12887-021-02542-1

**Published:** 2021-02-13

**Authors:** Silvestre Garcia-de la Puente, Karla A. Flores-Arizmendi, María J. Delgado-Montemayor, Tania T. Vargas-Robledo

**Affiliations:** 1 Department of Research Methodology, Instituto Nacional de Pediatría, Ciudad de México, Mexico; 2 Pediatrician. Down Syndrome Clinic, Instituto Nacional de Pediatría, Ciudad de México, Mexico

**Keywords:** Lipids profile, Dyslipidemia, Down syndrome, Children

## Abstract

**Introduction:**

Down syndrome (DS) is associated with various congenital anomalies and metabolic alterations, such as dyslipidemias, that can lead to cardiovascular disease in adulthood. This study was designed to describe the lipid concentrations and the frequency of dyslipidemias in children with DS.

**Materials and methods:**

The sample included 386 patients, 52.4% male. The study was carried out on children with DS, aged 2–18 years old, who were patients at the Mexican National Institute of Pediatrics between May 2016 and June 2017. Their height and weight were recorded, and their serum cholesterol, HDL cholesterol, and triglyceride levels were determined.

**Results:**

Of the total patients included, 57.5% had some type of dyslipidemia, 32.6% isolated and 24.9% combined. The most common alteration, considering both isolated and combined dyslipidemias, was low HDL, in 45.9%, followed by hypertriglyceridemia, in 26.2%. Among those with combined dyslipidemia, high TG with low HDL-c was the most common, in 17.9%. A significant association was found between dyslipidemia and obesity, as well as between dyslipidemia and central obesity. The percentiles of lipid values are reported.

**Conclusion:**

The presence of an unfavorable lipid profile is common in pediatric patients with Down syndrome, especially low HDL cholesterol and high triglycerides.

**Supplementary Information:**

The online version contains supplementary material available at 10.1186/s12887-021-02542-1.

## Introduction

Down syndrome (DS) is the most common human chromosomopathy, with a frequency of 1 in 700 live births [1]. It is associated with congenital deformities, illnesses, and metabolic abnormalities such as dyslipidemia, with a number of studies reporting abnormal lipid concentrations in this population [2–5]. Given the increasing life expectancy of people with DS, an unfavorable lipid profile can lead to cardiovascular disease in adulthood. There have been no studies on the subject in Mexico; the objective of the current study was thus to examine lipid profiles, and the frequency of dyslipidemia, in Mexican children and adolescents with DS.

## Materials and methods

A prospective, observational, cross-sectional study was designed of patients with Down syndrome, aged 2–18 years, at the Instituto Nacional de Pediatría (INP), during the period from May 2016 to June 2017. A total of 386 participants were included, of which 204 (52.8%) were male. Parents or guardians of participants provided written informed consent, and whenever possible participants themselves provided informed assent. Potential participants were excluded who had complex congenital cardiopathies, hypothyroidism under treatment, diabetes mellitus, nephrotic syndrome, chronic renal insufficiency, chronic malnutrition, or who were being treated with systemic steroids at the time.

Height and weight of patients at the INP Down syndrome clinic are measured routinely before each visit. Weight was measured with a Tanita BC-585F Fitscan Body Composition Monitor, in underwear, without shoes, to the nearest tenth of a kilogram. Height was measured with a Harpenden stadiometer, without shoes, in cm and mm. These measurements were used to calculate body mass index (BMI, kg/m^2^). Waist size was measured with a tape measure on the uncovered abdomen at the midpoint between the edge of the lower rib and the iliac crest, with the measurement taken after exhalation. The instruments were calibrated, and the personnel taking the measurements were standardized. All participants fasted at least 12 h before laboratory studies, which included the following: Hematic biometry, a general urine examination, and blood chemistry were taken. Total cholesterol (TC), HDL cholesterol (HDL-c), and triglycerides (TG) were analyzed using enzymatic methods, according to the manufacturer’s specifications, with a Beckman Coulter multiplier, DxC 700 AU; LDL cholesterol (LDL-c) was calculated with Friedewald’s formula: [6, 7] LDL = (CT-HDL-c-(TG/5)). Non-HDL cholesterol was calculated by subtracting HDL-c from CT.

Nutritional state was classified using the BMI percentile as follows: low weight, BMI < p5; normal nutritional state, BMI ≥ p5 and < p85; overweight, BMI ≥ p85 and < p95; obese, ≥ p95 [8, 9]. Central obesity was defined as a waist/height index > 0.50. Lipid values were classified according to the recommendations of the Expert Panel on Integrated Guidelines for Cardiovascular Health and Risk Reduction in Children and Adolescents of the National Heart, Lung, and Blood Institute, National Institutes of Health (NIH), proposed in 1992 and modified in recent guidelines [10]. Dyslipidemia was defined as the presence of one or more of the following conditions: TC ≥ 200 mg/dL, LDL-c ≥ 130 mg/dL, non-HDL cholesterol ≥145 mg/dL, HDL-c < 40 mg/dL, and TG ≥ 100 mg/dL in children under 10 years of age or TG ≥ 130 mg/dL in children 10 years or older. With these values, dyslipidemias were classified as isolated when an abnormal value was found (high TC, LDL-c, non-HDL, and TG; low HDL-c) or combined where there were two or more abnormal values. The latter were subdivided into a) high TG and low HDL-c with normal TC and/or LDL-c, b) high TC and/or LDL-c with high TG and normal HDL-c, or c) “other,” any combination of two or more alterations in lipids not classified as a) or b).

### Statistical analysis

The anthropometric data and lipid values are summarized with means and standard deviations, with percentiles of the lipid values for the age group 2–18 years as a whole, and also percentiles of triglyceride values for the age groups under 10 years and 10–18 years. The categorical values, including the frequency of dyslipidemia, are summarized with frequencies and percentages. The association of dyslipidemia with obesity and central obesity was evaluated with Pearson’s X^2^. Comparisons with the population values reported in Mexico and the United States were performed using a one-sample T test or binomial test. The correlation between lipid values and BMI was evaluated with Pearson’s correlation.

## Results

A total of 386 participants were recruited, aged 2–18 years, 204 (52.8%) of them male. The anthropometric data and lipid values are summarized in Table 1. The waists of only 130 were measured for the calculation of the waist/height index. Table 2 shows the lipid percentiles and Table 3 the categorical variables, including the proportion with acceptable, borderline, and abnormal lipid values; of note is that 45.9% had low HDL-c and 26.2% had high TG. Table 4 summarizes the types of dyslipidemias found: 222 (57.5%) had dyslipidemias, 126 of them (32.6%) isolated and (24.9%) combined. Of the latter, the most frequent was high TG and low HDL-c with normal TC and/or LDL-c, in 69 (17.9%). An association was found between dyslipidemia and the presence of obesity and of central obesity (Table 5), with a positive correlation of 0.333 between the BMI and triglycerides, p < 0.001.

**Table 1:** Anthropometric data and lipid values

VARIABLE	n	Mean	Standard Deviation
Age (years)	386	8.44	4.76
Weight (Kg)	386	25.03	14.35
Weight percentile	386	26.25	24.43
Height (cm)	386	112.29	22.17
Height percentile	386	32.89	28.48
BMI	386	18.1	3.8
BMI percentile	386	50.92	28.38
Waist (cm)	130	62.29	13.52
Waist/height index	130	0.55	0.09
Total cholesterol mg/dl, (mmol/L)	386	157.23 (4.07)	27.7
Triglycerides (all) mg/dl, (mmol/L)	386	93.52 (1.06)	47.75
Triglycerides 2–9 years mg/dl, (mmol/L)	254	83.01 (0.94)	35.57
Triglycerides 10–18 years mg/dl, (mmol/L)	132	113.76 (1.28)	60.25
HDL cholesterol mg/dL, (mmol/L)	386	41.69 (1.08)	10.16
LDL cholesterol mg/dL, (mmol/L)	386	96.62 (2.5)	23.26
NON-HDL cholesterol mg/dL, (mmol/L)	386	115.53 (2.99)	25.59

**Table 2:** Lipid Percentiles

Percentiles							
	5	10	25	50	75	90	95
Cholesterol mg/dL	112.7	120	138	158	176	192.3	203.3
mmol/L	2.91	3.1	3.57	4.09	4.55	4.97	5.26
Triglycerides all, mg/dL	43	49.7	63	83.5	115	145.3	173.2
mmol/L	0.49	0.56	0.71	0.94	1.3	1.64	1.96
Triglycerides 2–9 y, mg/dL	41	46	59	79	99.25	122	136.5
mmol/L	0.46	0.52	0.67	0.89	1.12	1.38	1.54
Triglycerides 10–18 y, mg/dL	50.95	58	72.25	99	131.75	182.7	265.15
mmol/L	0.58	0.65	0.82	1.12	1.49	2.06	2.99
LDL cholesterol mg/dL	59.64	66.97	80.1	96.65	112.7	126.26	135.69
mmol/L	1.54	1.73	2.07	2.5	2.91	3.27	3.51
HDL cholesterol mg/dL	26.91	29.87	34.6	41	47.63	55.7	59.99
mmol/L	O,7	0.77	0.9	1.06	1.23	1.44	1.55
Non-HDL cholesterol mg/dL	74.72	83.84	97.3	114.55	132.02	150.89	159
mmol/L	1.93	2.17	2. 52	2.96	3.41	3.90	4.11

**Table 3:** Categorical Variables for Lipids

VARIABLE	*n*	FREQUENCY	PERCENTAGE
SEX	386		
Male		204	52.8
Female		182	47.2
CYTOGENETICS	386		
Regular Trisomy		297	76.9
Mosaicism		18	4.7
Robertsonian Translocation		15	3.9
Not determined		56	14.5
NUTRITIONAL STATE	386		
Low weight		20	5.2
Normal		307	79.5
Overweight		41	10.6
Obese		18	4.7
CENTRAL OBESITY	130	93	71.5
TOTAL CHOLESTEROL	386		
Acceptable		259	67.1
Borderline		102	26.4
High		25	6.5
HDL CHOLESTEROL	386		
Acceptable		124	32.1
Borderline		85	22
Low		177	45.9
TRIGLYCERIDES	386		
Acceptable		171	44.3
Borderline		114	29.5
High		101	26.2
LDL CHOLESTEROL	386		
Acceptable		276	71.5
Borderline		80	20.7
High		30	7.8
NON-HDL CHOLESTEROL	386		
Acceptable		234	60.6
Borderline		105	27.2
High		47	12.2

**Table 4:** Classification of Dyslipidemias in the 386 Participants

VARIABLE	FREQUENCY	PERCENTAGE
NO DYSLIPIDEMIA	164	42.5
DISLIPIDEMIA	222	57.5
Isolated dyslipidemia	126	32. 6
Low HDL	101	26.1
Elevated triglycerides	20	5.2
Elevated cholesterol and/or LDL	5	1.3
Combined dyslipidemia	96	24.9
High TG and low HDL	69	17.9
High TG and high TC and/or LDL-c	19	4.9
Other combinations	8	2.1

**Table 5:** Association of Dyslipidemia with Obesity and Central Obesity

	DYSLIPIDIMIA					
	YES		NO		*p*	*ODDS RATIO*
OBESITY	15	83.3%	3	16.7%	0.023	3.89
CENTRAL OBESITY	62	66.7%	31	33.3%	0.029	2.36

## Discussion

People with Down syndrome have been found to have less favorable lipid profiles than those without this condition [2–5]. Because there is little data, it has not been possible to analyze the mechanism by which children with DS develop this unfavorable profile, but genetic influence could be one of the mechanisms involved. A locus has been found in chromosome 21, in the region 21q11, that carries the information for a receptor of very low density lipoproteins, and which probably plays an important role in the control mechanism of lipid metabolism in this group. A study carried out on fetuses with trisomy 21 found that they presented greater levels of cholesterol in utero than a control group [11]. A study by Buonuomo et al. [4] found that a high percentage of children with DS aged 2–18 years presented concentrations exceeding the recommendations of the Expert Panel on Integrated Guidelines for Cardiovascular Health and Risk Reduction in Children and Adolescents of the National Institutes of Health (NIH). High lipid levels in children with DS were reported in 1991 by Zamorano et al. [2] in a study carried out on 72 children with DS and a healthy control group, and in 2012 Adelekan et al. [5] confirmed that children with DS had a less favorable lipid profile than their healthy siblings. Our study found a high frequency of dyslipidemia, close to 60%, comparable to the 58.3% found by De la Piedra et al. [3] in a group of 218 Chilean children and adolescents with DS.

There are few studies of the frequency of dyslipidemia in Mexican children and adolescents. Bibiloni et al., [12] in a study of 451 typically developing children aged 2–10 years in the state of Nuevo León, found a frequency of 54.3%, similar to our result. Our participants, however, showed a greater frequency of hypoalphalipoproteinemia (low HDL-c), 45.9%, versus 26.8% in their study, while other types of isolated dyslipidemia were found more often in the typically developing children. We also find a greater frequency of hypoalphalipoproteinemia in our participants than in the population of Mexican-Americans in the U.S. (45.9% vs. 14.8%), and also a greater frequency of hypertriglyceridemia (26.2% vs. 12.4%), though no differences in other types of hyperlipidemia [13]. The comparisons were made according to the age group, considering the reference values of lipids in Nuevo León and in the U.S.

Our results show that the most prevalent type of dyslipidemia was hypoalphalipoproteinemia (low HDL-c), in 45.9% of participants; the most prevalent of the combined dyslipidemias was high TG with low HDL-c, in 17.9%. This prevalence of dyslipidemias is greater than that reported by De la Piedra for children with DS in Chile, [3] although the overall distribution of dyslipidemias is similar.

Low levels of HDL-c are generally associated with central obesity and are also related to tobacco use, physical inactivity, and diets low in monounsaturated fats. HDL-c is an excellent marker of the risk of arteriosclerosis and especially of coronary disease, and in a large number of epidemiological studies it is the lipid risk factor with the greatest predictive power: it is the most frequent lipid disorder in persons with premature coronary disease. When low HDL-c accompanies elevated LDL-c, that is, when the LDL-c/HDL-c quotient is greater than 5, the atherogenic risk is very high, and is doubled in the presence of hypertriglyceridemia [14]. Commonly associated medical conditions are diabetes mellitus, chronic kidney disease, and chronic inflammatory disorders like rheumatoid arthritis. Certain medications can reduce HDL-c levels, including beta blockers, thiazide-like diuretics, androgens, progestins, and probucol. The principal recommendations for treatment continue to be dietary; the effects of niacin (vitamin B3) on elevated levels of HDL-c have been studied [15].

There is no previous data on the frequency of overweight and obesity in the pediatric population with DS in Mexico. In our study, 10.6% of the participants were overweight and 4.7% obese, fewer than reported by Buonuomo et al. [4] There is also an important difference in the figures reported for overweight and obesity for the typically developing pediatric population of Mexico. According to the ENSANUT 2018 survey, [16] the prevalence of overweight in children aged 5–11 years is 18.1% and the prevalence of obesity is 17.5%. In adolescents aged 12–19 years, the figures are 14.6 and 23.8% respectively. Although some studies have found no correlation between the lipid profile of children with DS and their nutritional state, we found a positive correlation, with statistically significant associations between obesity and dyslipidemia (*p* = .023) and between central obesity and dyslipidemia (*p* = .029); we also found a positive correlation between triglyceride levels and BMI. In Mexico, Arjona-Villicaña et al., [17] in a study of 289 obese children in the state of Yucatán, found that 83.7% presented with dyslipidemia. Kavey, in a review article, mentions that combined dyslipidemia is the most prevalent lipid abnormality associated with obesity and is seen in 40% of obese adolescents [18]. Our results, by comparison, showed a low prevalence of obesity but a high prevalence of dyslipidemia.

In addition to having dyslipidemia and obesity, people with DS are often sedentary, malnourished, and are predisposed to diabetes mellitus: these factors should be considered potential comorbidities that increase the risk of cardiovascular disease. The increased lifespan of people with DS has also changed the incidence of chronic illnesses, which creates concern for their long-term health and in particular the appearance of non-transmissible diseases like cardiovascular atherosclerosis. In the past they were considered “free of atheromas,” but recent data from two large-scale studies of people with DS suggests that they may have a greater risk of mortality from ischemic cardiopathy and cerebrovascular disease than the general population [19, 20].

The most convincing evidence of the grouping of risk factors in young people is from Bogalusa’s autopsy study, which reports the relationship between hyperlipidemia in childhood and increased cardiovascular risk in adulthood, as well as a greater percentage of fatty streaks on the surface of the aortic intima [21]. These findings regarding coronary atherosclerosis in youth provide an additional justification for the evaluation of cardiovascular risk from an early age.

Although the current guidelines for monitoring the health of children with DS emphasize the importance of preventive interventions for different conditions, there are no specific recommendations with respect to scheduled testing of the lipid profile. We suggest a systematic analysis of lipids in regular health exams from the age of 2 years, as well as specific preventive strategies including a low-fat diet, increased physical activity, and a reduction in exposure to tobacco smoke. In Table 2, we include the percentiles of lipid values, which can be used as a reference, with therapeutic measures indicated for values above the 75th percentile for total cholesterol, triglycerides, LDL-c, and non-HDL-c, or below the 25th percentile for HDL-c.

There are no data demonstrating how cardiovascular comorbidities or risk factors in children with DS can affect the incidence of cardiovascular events; long-term monitoring is thus crucial to establish whether an unfavorable lipid profile can mean an increase in morbidity and mortality as the result of cardiovascular disease.

## Conclusions

The frequency of dyslipidemia is high in children and adolescents with DS, especially of hypoalphalipoproteinemia and hypertriglyceridemia. Routine evaluation of the lipid profile is recommended in this population from the age of 2 years.

## Supplementary Information

**Additional file 1.**

**Additional file 2.**

**Additional file 3.**

## Data Availability

All data generated or analyzed during this study are included in this published article [and its supplementary information files].
