# Supplementary material for: Lipid profile of Mexican children with Down syndrome
Source: BMC Pediatr. 2021 Feb 13;21:77. doi: 10.1186/s12887-021-02542-1 (PMC7881458; doi:10.1186/s12887-021-02542-1)
Supplement: Supplementary file 1 — Additional file 1. [file 12887_2021_2542_MOESM1_ESM.pdf]

Lipid profile of Mexican children with Down symdrome

Authors: Silvestre García de la Puente, Karla A. Flores Arizmendi, María J. Delgado Montemayor, Tania T. Vargas Robledo

sex	0 = female, 1 = male
age 2 a 9 años	0 = 2 a 9 years, 1 = 10 a 18 years
age 4 groups	0 = 2 a 4, 1 = 5 a 7, 2 = 8 a 10, 3 = 11 a 18 years
age 3 groups	0 = 2 a 7, 1 = 8 a 10, 2 = 11 a 18 years
age 11 a 18	0 = 2 a 10, 1 = 11 a 18 years
overweight_obesity	0 = no, 1 = yes
nutrition	0 = low weight, 1 = normal, 2 = overweight, 3 = obesity
chol classific	0 = acceptable, 1 = borderline, 2 = high
LDL classific	0 = acceptable, 1 = borderline, 2 = high
TG classific	0 = acceptable, 1 = borderline, 2 = high
HDL classific	0 = acceptable, 1 = borderline, 2 = low
noHDL clasific	0 = acceptable, 1 = borderline, 2 = high
dyslipidemia classification	0 = normal, 1 = high cholesterol and/or LDL, 2 = high triglycerid, 3 = HDL low
	4 = combined dyslipidemia
high chol and low HDL	1 = high cholesterol and low hdl
simple classif	0 = without dyslipidemia, 1 = isolated dyslipidemia, 2 = combined dyslipidemia
nutrition 2	0 = low weight or normal, 1 = overweight, 2 = obesity
all dichotomous variables	0 = no, 1 = yes
