# Supplementary material for: Lipid profile of Mexican children with Down syndrome
Source: BMC Pediatr. 2021 Feb 13;21:77. doi: 10.1186/s12887-021-02542-1 (PMC7881458; doi:10.1186/s12887-021-02542-1)
Supplement: Supplementary file 2 — Additional file 2. [file 12887_2021_2542_MOESM2_ESM.pdf]

Lipid profile of Mexican children with Down symdrome

Authors: Silvestre García de la Puente, Karla A. Flores Arizmendi, María J. Delgado Montemayor, Tania T. Vargas Roble

ID	cytogenetics	weight Kg	percentile weight	height cm	percentile height	sex	age year
363	REGULAR	10.6	50	80	50	0	
310	REGULAR	10.18	25	79	25	0	
300	REGULAR	9.535	10	76.8	10	1	
266	REGULAR	9.46	10	79	25	1	
330	REGULAR	10.4	25	79	25	1	
133	NOT SPECIFIED	7.4	2	72		0	
379	REGULAR	10.3	25	82	75	0	
61	REGULAR	10.3	25	79.7	25	1	
69	REGULAR	9.885	2	76	5	1	
308	REGULAR	11.4	50	86.5	98	0	
273	REGULAR	10.6	50	81	50	0	
121	REGULAR	12.7	75	84.5	75	1	
323	NOT SPECIFIED	11.5	50	85.5	75	1	
48	REGULAR	12.7	75	86	75	1	
355	REGULAR	7.68	2	76.6	5	1	
9	REGULAR	12.78	75	84	75	1	
65	REGULAR	8.3	2	75	10	0	
317	MOSAICISM	9.2	10	79	25	0	
114	REGULAR	8.3	2	73.9	5	0	
125	REGULAR	8.43	2	76.4	5	1	
230	REGULAR	8.55	2	82.7	50	1	
98	MOSAICISM	10.48	25	84.6	50	0	
286	REGULAR	10.4	25	82	25	0	
328	REGULAR	11.98	50	87	75	0	
45	REGULAR	11.7	25	86	50	1	
165	REGULAR	9.735	5	69	2	1	
374	REGULAR	10	10	78.7	5	1	
126	NOT SPECIFIED	9.7	5	79	5	1	
74	REGULAR	14	75	97	98	1	
333	REGULAR	10.88	10	82	25	1	
380	REGULAR	9.8	5	80	10	1	
356	REGULAR	10.6	10	78.5	5	1	
221	REGULAR	12.82	75	80	25	0	
12	REGULAR	9	2	85	75	0	
321	REGULAR	10.92	25	84	50	0	
73	REGULAR	11	25	84.7	50	0	
176	REGULAR	10.29	10	83.1	25	1	
18	NOT SPECIFIED	13.3	75	95	98	1	
316	MOSAICISM	10.5	25	82	25	0	
331	REGULAR	8.835	2	76.4	5	0	
283	REGULAR	10.3	25	80.7	25	0	

255	REGULAR	10.06	25	83	50	0
319	REGULAR	9.52	2	79	5	1
123	REGULAR	10.82	10	84	25	1
1	REGULAR	13.6	75	88	75	1
291	REGULAR	12.55	50	90	90	1
154	REGULAR	9.6	5	76	2	1
318	REGULAR	13.01	75	88	75	1
152	REGULAR	14.25	90	90	98	0
91	REGULAR	12.53	50	90.5	90	1
274	REGULAR	11.8	25	80.3	10	1
26	REGULAR	10.8	10	89	75	1
220	REGULAR	11.1	10	86.3	50	1
85	REGULAR	10.3	10	83	25	0
371	REGULAR	9.6	2	80	2	1
287	REGULAR	10.7	10	87	50	0
295	REGULAR	10.7	10	87	50	0
171	NOT SPECIFIED	13.25	50	88.5	50	1
322	REGULAR	13.25	50	89	50	1
352	REGULAR	13	50	88	50	1
189	TRANSLOCATION	10.85	10	80	10	0
243	REGULAR	11.96	25	85	25	0
14	REGULAR	14.2	75	93.3	98	0
87	REGULAR	10	5	86.1	50	0
36	REGULAR	11.44	10	85	25	1
151	REGULAR	13.3	50	92	75	1
233	REGULAR	11.23	10	88.7	50	1
138	NOT SPECIFIED	14.1	75	98	98	1
153	TRANSLOCATION	10.5	5	85	10	0
166	REGULAR	11.93	10	87.9	25	1
343	TRANSLOCATION	12.8	25	87	25	0
149	REGULAR	14.2	50	93	75	0
200	REGULAR	10.6	5	81.2	2	1
278	REGULAR	10.5	5	87.5	25	0
271	REGULAR	12.34	10	88	25	1
17	REGULAR	11.31	10	83	2	1
64	REGULAR	11.262	10	95	75	1
49	REGULAR	13	25	88	25	1
110	REGULAR	21.5	98	103	98	1
213	NOT SPECIFIED	16.1	75	92	75	0
307	MOSAICISM	14.3	50	89.5	50	0
127	REGULAR	14	50	93	75	0
216	NOT SPECIFIED	12.5	10	86.4	10	1
72	TRANSLOCATION	12.01	10	89.1	25	1
272	REGULAR	13.3	25	90.5	25	0
6	REGULAR	10.69	2	85.5	5	0

40	REGULAR	11.85	5	97	75	1
368	REGULAR	16.2	75	96.5	75	1
222	REGULAR	15.6	50	92.6	25	1
325	REGULAR	12.4	10	88	10	0
115	REGULAR	13.9	50	93.5	50	0
288	REGULAR	14.1	25	92	25	1
338	REGULAR	21.15	98	107	98	1
251	NOT SPECIFIED	15.6	50	90.5	25	1
70	REGULAR	12.45	10	88	10	1
109	REGULAR	10.6	2	88	10	0
146	REGULAR	11.58	5	93	50	1
181	REGULAR	12.8	25	89	25	0
53	REGULAR	12.9	10	93	25	0
265	REGULAR	13.79	10	98	50	1
314	NOT SPECIFIED	15.2	25	98	50	1
339	TRANSLOCATION	19.7	90	102	90	0
186	REGULAR	17.5	75	100	75	0
33	REGULAR	13.15	10	94	25	1
38	REGULAR	14.5	25	102	90	0
170	REGULAR	13.7	25	99	75	0
185	REGULAR	18.5	75	100.5	75	0
305	REGULAR	14.4	25	94	25	0
128	REGULAR	12.95	10	90	10	0
239	MOSAICISM	20	90	101	75	0
7	REGULAR	16	50	101.5	90	0
108	NOT SPECIFIED	17.4	75	94	25	0
320	MOSAICISM	13.2	10	95	25	0
377	NOT SPECIFIED	16.1	50	96.6	50	0
82	REGULAR	14.5	25	101	75	1
347	REGULAR	14.7	25	95.5	25	1
5	REGULAR	15.75	50	96	50	1
182	REGULAR	14	25	97.5	50	1
284	REGULAR	10.49	2	86	2	1
129	REGULAR	15.1	25	95	25	0
327	REGULAR	15.6	25	100	50	1
46	NOT SPECIFIED	16	25	105	75	1
60	REGULAR	13.45	5	96	10	1
290	REGULAR	14.5	10	89.5	2	1
210	REGULAR	13	5	97	25	0
86	REGULAR	12.8	5	91	5	0
164	REGULAR	11.45	2	89.4	2	1
39	REGULAR	14.3	10	96.3	25	0
209	REGULAR	15.3	25	97	25	0
119	REGULAR	15.2	25	92	5	1
334	REGULAR	18.15	50	105.5	75	1

285	NOT SPECIFIED	16.5	25	99	25	1
4	REGULAR	16.8	50	106	90	1
30	REGULAR	13.2	5	96	25	1
250	REGULAR	18	50	110	95	0
226	REGULAR	14.6	25	101	50	0
158	REGULAR	20	75	104.5	50	0
336	REGULAR	15.7	10	99	5	1
235	REGULAR	15.2	10	97	10	1
113	REGULAR	15.65	10	102	25	1
204	REGULAR	24.5	98	107	75	1
124	REGULAR	12.7	2	98.2	10	1
35	REGULAR	15.5	10	103	50	1
229	REGULAR	13.66	2	94.2	2	1
202	REGULAR	17.25	25	101	25	1
173	REGULAR	18.2	50	103.5	50	1
309	REGULAR	17.6	50	97	10	0
159	REGULAR	13	2	94	2	1
280	REGULAR	18.35	50	107	75	1
191	TRANSLOCATION	21.35	75	113	98	1
97	REGULAR	15.9	10	101.5	25	1
134	REGULAR	20.1	75	112	95	1
337	TRANSLOCATION	21	75	114	90	1
54	NOT SPECIFIED	17.1	10	106	50	1
55	REGULAR	17.1	25	106	50	0
184	REGULAR	14	2	101	25	0
364	REGULAR	18.3	50	105	50	0
262	REGULAR	19.2	50	103	25	0
324	REGULAR	15.8	10	100	10	0
197	REGULAR	18.2	50	104.5	50	0
232	MOSAICISM	21.35	50	108	50	1
349	NOT SPECIFIED	26.5	95	112	75	1
130	REGULAR	12.95	2	90	2	1
21	TRANSLOCATION	16.5	5	104.3	10	1
41	REGULAR	17.1	10	104	25	0
116	REGULAR	14.9	2	107	50	0
68	REGULAR	17.2	10	98.2	5	0
10	REGULAR	19.5	25	119	95	1
62	REGULAR	20.05	50	118	90	1
88	REGULAR	16	5	108	25	1
42	REGULAR	17.3	10	103.5	10	1
107	REGULAR	15.2	5	102	10	0
297	REGULAR	17.9	10	102	5	1
231	REGULAR	16.5	5	107.4	25	1
228	REGULAR	20.3	50	114	90	0
329	REGULAR	17.3	10	105.5	25	0

8	REGULAR	19.115	10	104.9	10	1
22	REGULAR	14.3	2	105.9	10	0
245	REGULAR	19.3	25	105.2	10	0
386	NOT SPECIFIED	15.35	2	98	2	1
193	REGULAR	24	50	117.5	75	1
76	REGULAR	18.2	10	99.4	2	1
177	REGULAR	17.2	5	109	10	1
112	REGULAR	21.6	25	106	10	1
156	REGULAR	22.7	25	110	10	1
275	REGULAR	23	50	114	50	0
101	REGULAR	20.75	25	110	25	0
258	REGULAR	25.15	50	117	75	0
253	REGULAR	19.85	10	108	5	1
155	NOT SPECIFIED	16.8	2	106	2	1
203	REGULAR	21.2	25	108	25	0
59	REGULAR	20.2	25	111	25	0
117	TRANSLOCATION	19.8	10	110	10	1
94	REGULAR	17.5	5	101.5	2	1
306	REGULAR	17.8	5	105	10	0
311	REGULAR	20	25	106	10	0
303	TRANSLOCATION	18.2	5	110.5	10	1
136	NOT SPECIFIED	16	2	106.5	2	1
277	NOT SPECIFIED	16	2	95	2	1
225	REGULAR	17	2	111	25	0
56	REGULAR	24	50	107	5	1
206	REGULAR	25	50	123	75	1
132	REGULAR	18	5	105	2	1
282	REGULAR	19.45	10	110	10	1
373	MOSAICO	17.5	2	110	5	1
51	TRANSLOCATION	22.2	25	118	25	1
199	NOT SPECIFIED	25.5	50	110.3	10	0
144	REGULAR	21.4	25	116.8	50	0
83	REGULAR	27.7	75	115	50	0
111	REGULAR	20	10	120.7	50	1
66	REGULAR	18.1	2	112.9	10	1
89	REGULAR	19	5	112.5	25	0
162	REGULAR	20	5	109.4	10	0
335	REGULAR	16.2	2	109	5	0
289	REGULAR	23	10	118	25	1
299	REGULAR	24.85	25	116	10	1
215	REGULAR	21.2	10	114	25	0
142	REGULAR	21	5	115	10	1
301	REGULAR	26.2	25	117.5	25	1
384	REGULAR	21	5	115	5	1
192	REGULAR	28.2	50	118	10	1

217	REGULAR	19.3	2	120	25	0	
212	REGULAR	15	2	102	2	1	
207	MOSAICISM	24.2	10	119	10	1	
93	REGULAR	20.6	5	119	25	0	
261	REGULAR	27.22	25	122	50	0	
163	REGULAR	25.5	10	126	50	1	
78	REGULAR	24.6	25	115	10	0	
270	REGULAR	19.55	2	114	10	0	
292	NOT SPECIFIED	22.8	10	116	10	1	
67	REGULAR	24.65	10	111.5	2	1	
52	NOT SPECIFIED	25.4	10	126	75	0	
237	TRANSLOCATION	30	50	127	75	0	
24	REGULAR	25.45	10	127	75	0	
175	REGULAR	28.3	50	120	25	0	
179	REGULAR	25.2	10	119	10	1	
15	REGULAR	35.6	75	125	50	1	
90	REGULAR	25	10	122	25	1	
256	MOSAICISM	28	50	126	50	1	
141	REGULAR	20.6	2	109.3	2	0	
99	REGULAR	31	50	127	50	0	
120	REGULAR	26.7	25	121.5	25	0	
267	REGULAR	22.7	10	119	10	0	
367	REGULAR	23	10	118	10	1	
80	REGULAR	30.3	50	125	50	0	
342	REGULAR	20.3	2	119.9	10	0	
71	NOT SPECIFIED	30.1	50	126	50	1	
241	REGULAR	34	50	127	50	1	
201	REGULAR	26.1	25	122	25	1	
58	REGULAR	27	25	114	2	1	
3	REGULAR	30.8	50	137	95	0	
214	REGULAR	22.75	10	114	2	1	
254	REGULAR	23.15	10	125.6	25	1	
139	NOT SPECIFIED	40.4	75	133	75	1	
351	REGULAR	43.5	75	135	75	0	
104	REGULAR	23.15	5	111	2	1	
122	REGULAR	22.05	2	124	25	0	[illegible]
32	REGULAR	24.85	10	122.5	10	1	[illegible]
118	REGULAR	25.35	10	125	25	0	[illegible]
180	NOT SPECIFIED	25	10	116	2	0	[illegible]
269	REGULAR	21.7	2	122	5	0	[illegible]
259	REGULAR	39.7	75	131.4	25	0	[illegible]
246	REGULAR	23.4	2	113.8	2	0	[illegible]
16	REGULAR	35.6	50	136.5	75	0	[illegible]
77	REGULAR	37	50	135	75	1	[illegible]
244	NOT SPECIFIED	20.9	2	107.8	2	1	[illegible]

84	REGULAR	33.65	50	125	10	0	[illegible]
315	REGULAR	33.8	50	128	25	0	[illegible]
190	NOT SPECIFIED	44.6	75	132.5	50	0	[illegible]
23	REGULAR	26.3	5	124	10	0	[illegible]
178	REGULAR	26.3	10	124	10	0	[illegible]
37	REGULAR	22.3	2	123.5	10	1	[illegible]
247	REGULAR	39.8	50	132.5	25	0	
140	REGULAR	32	25	126.5	10	1	
234	REGULAR	28.8	10	124.5	10	1	[illegible]
302	REGULAR	27.3	10	126	10	0	[illegible]
205	REGULAR	49	75	134.5	50	0	[illegible]
312	REGULAR	33.6	25	123.2	5	0	[illegible]
219	REGULAR	32.6	25	129	10	0	[illegible]
2	REGULAR	28.65	10	130	25	0	[illegible]
350	REGULAR	28.6	10	124	5	0	[illegible]
240	NO ESPECIFICA	23	2	130	25	1	[illegible]
224	REGULAR	37.1	50	137	75	1	[illegible]
187	REGULAR	42.5	75	136.5	50	1	[illegible]
135	REGULAR	37.05	50	132	25	0	[illegible]
27	REGULAR	39.6	50	130	10	0	[illegible]
268	REGULAR	41.5	50	130	10	0	[illegible]
260	REGULAR	41.2	50	142.9	75	1	[illegible]
211	NOT SPECIFIED	30	10	128.5	10	1	[illegible]
44	NOT SPECIFIED	24.2	2	129	10	1	[illegible]
198	MOSAICISM	40.135	50	147	95	1	[illegible]
276	NOT SPECIFIED	28.75	10	119	2	0	[illegible]
103	REGULAR	36	25	130	10	1	[illegible]
194	REGULAR	37.2	25	137	50	1	[illegible]
75	REGULAR	28.2	5	132	10	0	
168	REGULAR	31.95	10	134	25	1	
172	NOT SPECIFIED	33.8	25	144	75	1	
188	NOT SPECIFIED	35	25	147	90	1	[illegible]
31	REGULAR	34.4	25	141	50	0	[illegible]
227	NOT SPECIFIED	29.9	10	130	10	0	[illegible]
19	NOT SPECIFIED	41.4	50	139	50	0	[illegible]
238	REGULAR	42.5	50	145	75	1	[illegible]
160	NOT SPECIFIED	34.3	25	147	90	1	[illegible]
345	REGULAR	39.5	25	128	2	1	[illegible]
346	REGULAR	31	10	132	10	1	[illegible]
157	REGULAR	37.8	25	138.5	25	0	[illegible]
344	REGULAR	34.75	10	132	10	0	[illegible]
196	NOT SPECIFIED	35.5	25	130	2	1	[illegible]
252	REGULAR	30	5	131	10	0	[illegible]
293	REGULAR	33.5	10	132	10	0	[illegible]
92	REGULAR	29.8	5	129	2	1	[illegible]

11	REGULAR	50	50	146	75	0	
279	REGULAR	43.6	50	141.6	50	0	[illegible]
102	REGULAR	52.5	75	142.5	25	1	[illegible]
28	NOT SPECIFIED	53.4	75	138.4	25	0	[illegible]
218	REGULAR	35	10	138	10	1	[illegible]
195	TRANSLOCATION	48	50	142	10	1	[illegible]
43	REGULAR	37.9	10	147.5	50	1	[illegible]
294	REGULAR	43.3	25	143.4	25	1	[illegible]
50	REGULAR	53.1	50	153	75	1	[illegible]
81	NOT SPECIFIED	44.5	25	140	25	0	
381	REGULAR	47.5	25	142	10	1	[illegible]
150	REGULAR	48.3	50	144	50	0	[illegible]
313	REGULAR	32.2	5	125	2	0	[illegible]
100	NOT SPECIFIED	48.7	50	142.8	50	0	[illegible]
143	NOT SPECIFIED	35.65	10	142.5	50	0	[illegible]
63	MOSAICISM	38.5	10	135	10	0	[illegible]
296	MOSAICISM	38.5	10	135	10	0	[illegible]
147	REGULAR	33	2	130	5	0	[illegible]
304	NOT SPECIFIED	30.5	2	134	10	0	[illegible]
105	REGULAR	45.3	25	148	75	0	[illegible]
96	NOT SPECIFIED	80.2	90	157	75	1	[illegible]
47	REGULAR	48.5	25	135	2	1	[illegible]
263	REGULAR	52	25	147	25	1	[illegible]
332	REGULAR	58.75	50	156	50	1	[illegible]
131	REGULAR	52.6	50	139	25	0	[illegible]
354	REGULAR	47.5	10	137.5	2	1	[illegible]
353	REGULAR	62.3	50	157	50	1	[illegible]
360	NOT SPECIFIED	51	25	144	10	1	[illegible]
208	REGULAR	40.5	5	146	10	1	[illegible]
264	MOSAICISM	66.9	75	159	75	1	[illegible]
106	REGULAR	28.1	2	137.6	10	0	[illegible]
183	NOT SPECIFIED	47	25	135	10	0	[illegible]
34	REGULAR	45.65	25	143.5	50	0	[illegible]
223	MOSAICISM	42.5	10	131	2	0	[illegible]
25	TRANSLOCATION	42.8	5	148	10	1	[illegible]
249	REGULAR	47.8	25	147.5	75	0	[illegible]
161	REGULAR	41	10	142	25	0	[illegible]
95	REGULAR	57.5	50	143.8	50	0	[illegible]
281	REGULAR	44.9	10	132	5	0	[illegible]
236	REGULAR	75	75	157	50	1	[illegible]
348	REGULAR	49	10	154	25	1	[illegible]
79	MOSAICISM	45.7	5	145	5	1	[illegible]
145	REGULAR	51.55	10	144	5	1	[illegible]
248	NOT SPECIFIED	69.6	75	153.3	25	1	[illegible]
242	NOT SPECIFIED	39.3	5	146.7	50	0	[illegible]

174	NOT SPECIFIED	50.05	10	144.3	5	1	[illegible]
340	REGULAR	51	10	125	2	1	[illegible]
341	NOT SPECIFIED	66	50	156	50	1	[illegible]
383	NOT SPECIFIED	58.7	25	145	5	1	[illegible]
169	REGULAR	33.1	2	137	10	0	[illegible]
13	REGULAR	49.55	25	141	25	0	[illegible]
378	REGULAR	39.1	5	138	10	0	[illegible]
137	REGULAR	49.55	25	142.3	25	0	[illegible]
376	NOT SPECIFIED	40.4	5	141	25	0	[illegible]
148	REGULAR	46	10	136	10	0	[illegible]
29	NOT SPECIFIED	47	2	143	2	1	[illegible]
20	NOT SPECIFIED	43	10	148.3	75	0	[illegible]
57	NOT SPECIFIED	41.45	2	141	2	1	[illegible]
382	REGULAR	59.9	25	155	25	1	[illegible]
167	REGULAR	39.2	5	127	2	0	[illegible]
365	REGULAR	45.7	10	132	5	0	[illegible]
361	REGULAR	44.7	10	134	5	0	
369	REGULAR	41	5	131	2	0	
372	REGULAR	41	5	146	50	0	
358	REGULAR	44.4	10	141.5	25	0	
375	REGULAR	43.1	5	137.6	10	0	
385	MOSAICISM	60	50	142	25	0	
362	REGULAR	48.65	10	134.5	5	0	
370	NOT SPECIFIED	46	10	142	25	0	
357	REGULAR	60.5	50	148.6	75	0	
366	REGULAR	62.7	50	139.5	25	0	
257	MOSAICISM	40	2	146	5	1	
359	REGULAR	48.8	5	152	25	1	
298	REGULAR	49	5	154.6	25	1	
326	TRANSLOCATION	57.8	25	144	2	1	

age 2_9	age 4 groups	age 3 groups	age 11_18	IMC	percentile IMC	overweight_obesity	nutr
0	0	0	0	16.5625	75	0	
0	0	0	0	16.3114885	75	0	
0	0	0	0	16.1658393	50	0	
0	0	0	0	15.1578273	50	0	
0	0	0	0	16.6639962	50	0	
0	0	0	0	14.2746914	10	0	
0	0	0	0	15.3182629	50	0	
0	0	0	0	16.2151355	50	0	
0	0	0	0	17.1139197	75	0	
0	0	0	0	15.2360587	50	0	
0	0	0	0	16.1560738	75	0	
0	0	0	0	17.7864921	75	0	
0	0	0	0	15.7313361	50	0	
0	0	0	0	17.171444	75	0	
0	0	0	0	13.088916	5	0	
0	0	0	0	18.1122449	85	1	
0	0	0	0	14.7555556	25	0	
0	0	0	0	14.7412274	25	0	
0	0	0	0	15.1980971	50	0	
0	0	0	0	14.4424495	10	0	
0	0	0	0	12.5012976	5	0	
0	0	0	0	14.6426795	10	0	
0	0	0	0	15.4669839	50	0	
0	0	0	0	15.8277183	50	0	
0	0	0	0	15.8193618	50	0	
0	0	0	0	20.447385	95	1	
0	0	0	0	16.1454642	50	0	
0	0	0	0	15.542381	50	0	
0	0	0	0	14.8793708	25	0	
0	0	0	0	16.1808447	50	0	
0	0	0	0	15.3125	25	0	
0	0	0	0	17.2015092	75	0	
0	0	0	0	20.03125	95	1	
0	0	0	0	12.4567474	5	0	
0	0	0	0	15.4761905	50	0	
0	0	0	0	15.3329551	50	0	
0	0	0	0	14.9009284	25	0	
0	0	0	0	14.7368421	25	0	
0	0	0	0	15.6157049	50	0	
0	0	0	0	15.1363038	50	0	
0	0	0	0	15.8157632	50	0	
0	0	0	0	14.6029903	25	0	

0	0	0	0	15.2539657	25	0
0	0	0	0	15.3344671	25	0
0	0	0	0	17.5619835	90	1
0	0	0	0	15.4938272	25	0
0	0	0	0	16.6204986	50	0
0	0	0	0	16.8001033	75	0
0	0	0	0	17.5925926	90	1
0	0	0	0	15.2986783	25	0
0	0	0	0	18.2999927	90	1
0	0	0	0	13.6346421	5	0
0	0	0	0	14.9039501	25	0
0	0	0	0	14.9513718	10	0
0	0	0	0	15	25	0
0	0	0	0	14.1366099	10	0
0	0	0	0	14.1366099	10	0
0	0	0	0	16.9172332	75	0
0	0	0	0	16.7276859	75	0
0	0	0	0	16.7871901	75	0
0	0	0	0	16.953125	75	0
0	0	0	0	16.5536332	75	0
0	0	0	0	16.3126702	75	0
0	0	0	0	13.4894331	10	0
0	0	0	0	15.83391	25	0
0	0	0	0	15.7136106	50	0
0	0	0	0	14.273567	10	0
0	0	0	0	14.6813828	10	0
0	0	0	0	14.532872	10	0
0	0	0	0	15.4405474	50	0
0	0	0	0	16.9110847	75	0
0	0	0	0	16.418083	75	0
0	0	0	0	16.0765852	50	0
0	0	0	0	13.7142857	10	0
0	0	0	0	15.9349174	50	0
0	0	0	0	16.4174771	50	0
0	0	0	0	12.4786704	5	0
0	0	0	0	16.7871901	75	0
0	0	0	0	20.265812	95	1
0	0	0	0	19.0217391	95	1
0	0	0	0	17.852127	85	1
0	0	0	0	16.1868424	75	0
0	0	0	0	16.7448988	75	0
0	0	0	0	15.1282119	25	0
0	0	0	0	16.2388205	50	0
0	0	0	0	14.6233029	25	0
0	0	0	0	12.5943246	5	0

0	0	0	0	17.3964402	85	1
0	0	0	0	18.192929	90	1
0	0	0	0	16.0123967	50	0
0	0	0	0	15.899797	50	0
0	0	0	0	16.6587902	75	0
0	0	0	0	18.4732291	90	1
0	0	0	0	19.0470376	95	1
0	0	0	0	16.0769628	50	0
0	0	0	0	13.6880165	10	0
0	0	0	0	13.3888311	5	0
0	0	0	0	16.1595758	50	0
0	0	0	0	14.9150191	25	0
0	0	0	0	14.3586006	10	0
0	0	0	0	15.8267389	50	0
0	0	0	0	18.935025	95	1
0	0	0	0	17.5	85	1
0	0	0	0	14.8822997	25	0
0	0	0	0	13.9369473	10	0
0	0	0	0	13.9781655	10	0
0	0	0	0	18.3163783	90	1
0	0	0	0	16.296967	50	0
0	0	0	0	15.9876543	50	0
0	0	0	0	19.605921	95	1
0	0	0	0	15.530588	50	0
0	0	0	0	19.6921684	95	1
0	0	0	0	14.6260388	25	0
0	0	0	0	17.2532781	75	0
0	0	0	0	14.2142927	5	0
0	0	0	0	16.1179792	50	0
0	0	0	0	17.0898438	75	0
0	0	0	0	14.7271532	25	0
0	0	0	0	14.1833423	5	0
0	1	0	0	16.7313019	75	0
0	1	0	0	15.6	50	0
0	1	0	0	14.5124717	25	0
0	1	0	0	14.594184	25	0
0	1	0	0	18.1018071	85	1
0	1	0	0	13.8165586	10	0
0	1	0	0	15.4570704	50	0
0	1	0	0	14.3261815	25	0
0	1	0	0	15.4199677	50	0
0	1	0	0	16.2610267	75	0
0	1	0	0	17.9584121	85	1
0	1	0	0	16.3069113	75	0
0	1	0	0	16.8350168	75	0

0	1	0	0	14.9519402	25	0
0	1	0	0	14.3229167	25	0
0	1	0	0	14.8760331	25	0
0	1	0	0	14.3123223	25	0
0	1	0	0	18.314599	75	0
0	1	0	0	16.0187736	50	0
0	1	0	0	16.1547455	75	0
0	1	0	0	15.0422914	25	0
0	1	0	0	21.3992488	95	1
0	1	0	0	13.1698475	5	0
0	1	0	0	14.6102366	25	0
0	1	0	0	15.3939082	50	0
0	1	0	0	16.9101069	75	0
0	1	0	0	16.9898947	75	0
0	1	0	0	18.7054947	85	1
0	1	0	0	14.7125396	25	0
0	1	0	0	16.0276007	75	0
0	1	0	0	16.7201817	75	0
0	1	0	0	15.4335218	50	0
0	1	0	0	16.0235969	50	0
0	1	0	0	16.1588181	75	0
0	1	0	0	15.2189391	50	0
0	1	0	0	15.2189391	25	0
0	1	0	0	13.7241447	10	0
0	1	0	0	16.5986395	75	0
0	1	0	0	18.0978415	85	1
0	1	0	0	15.8	50	0
0	1	0	0	16.6662851	75	0
0	1	0	0	18.3041838	85	1
0	1	0	0	21.1256378	95	1
0	1	0	0	15.9876543	50	0
0	1	0	0	15.1675462	25	0
0	1	0	0	15.8099112	50	0
0	1	0	0	13.0142371	10	0
0	1	0	0	17.8363289	75	0
0	1	0	0	13.770214	10	0
0	1	0	0	14.3995978	10	0
0	1	0	0	13.7174211	10	0
0	1	0	0	16.1497351	50	0
0	1	0	0	14.6097655	25	0
0	1	0	0	17.2049212	75	0
0	1	0	0	14.3045889	10	0
0	1	0	0	15.6201908	25	0
0	1	0	0	15.5432268	25	0
0	1	0	0	17.3709402	75	0

0	1	0	0	12.7509962	5	0
0	1	0	0	17.4391707	75	0
0	1	0	0	15.9829238	50	0
0	1	0	0	17.3834314	75	0
0	1	0	0	18.4203814	85	1
0	1	0	0	14.4768959	25	0
0	1	0	0	19.2239231	90	1
0	1	0	0	18.7603306	90	1
0	1	0	0	17.6977532	75	0
0	1	0	0	17.1487603	75	0
0	1	0	0	18.3724158	90	1
0	1	0	0	17.0181756	75	0
0	1	0	0	14.9519402	25	0
0	1	0	0	18.175583	85	1
0	1	0	0	16.3947732	50	0
0	1	0	0	16.3636364	50	0
0	1	0	0	16.9865806	75	0
0	1	0	0	16.1451247	50	0
0	1	0	0	17.7999288	75	0
0	1	0	0	14.9055097	25	0
0	1	0	0	14.1065485	10	0
0	1	0	0	17.7285319	75	0
0	1	0	0	13.7975814	5	0
0	1	0	0	20.9625295	95	1
0	1	0	0	16.5245555	50	0
0	1	0	0	16.3265306	50	0
0	1	0	0	16.0743802	50	0
0	2	1	0	14.4628099	10	0
0	2	1	0	15.9436943	25	0
0	2	1	0	20.9598976	85	1
0	2	1	0	15.6865735	25	0
0	2	1	0	20.9451796	85	1
0	2	1	0	13.7282587	5	0
0	2	1	0	14.2000767	5	0
0	2	1	0	15.0123457	25	0
0	2	1	0	16.7107273	50	0
0	2	1	0	13.6352159	5	0
0	2	1	0	16.5182419	50	0
0	2	1	0	18.4675981	75	0
0	2	1	0	16.3127116	50	0
0	2	1	0	15.879017	25	0
0	2	1	0	18.9769126	85	1
0	2	1	0	15.879017	25	0
0	2	1	0	20.2528009	75	0
0	2	1	0	13.4027778	5	0

0	2	1	0	14.4175317	10	0
0	2	1	0	17.0891886	50	0
0	2	1	0	14.5469953	10	0
0	2	1	0	18.2880946	75	0
0	2	1	0	16.0619803	25	0
0	2	1	0	18.6011342	75	0
0	2	1	0	15.0430902	10	0
0	2	1	0	16.9441141	50	0
0	2	1	0	19.8274649	75	0
0	2	1	0	15.9989922	25	0
0	2	1	0	18.6000372	75	0
0	2	1	0	15.7790316	25	0
0	2	1	0	19.6527778	75	0
0	2	1	0	17.7953534	50	0
0	2	1	0	22.784	90	1
0	2	1	0	16.7965601	50	0
0	2	1	0	17.6366843	50	0
0	2	1	0	17.2435586	50	0
0	2	1	0	19.2200384	75	0
0	2	1	0	18.0866738	75	0
0	2	1	0	16.0299414	25	0
0	2	1	0	16.5182419	50	0
0	2	1	0	19.392	75	0
0	2	1	0	14.120747	5	0
0	2	1	0	18.9594356	75	0
0	2	1	0	21.0800422	85	1
0	2	1	0	17.5356087	50	0
0	2	1	0	20.7756233	75	0
0	2	1	0	16.4100378	50	0
0	2	1	0	17.5053863	50	0
0	2	1	0	14.674784	10	0
0	2	1	0	22.8390525	90	1
1	2	1	0	23.8683128	95	1
1	2	1	0	18.7890593	75	0
1	2	1	0	14.3405307	10	0
1	2	1	0	16.5597668	25	0
1	2	1	0	16.224	25	0
1	2	1	0	18.5790725	75	0
1	2	1	0	14.5794141	10	0
1	2	1	0	22.993219	75	0
1	2	1	0	18.0688841	50	0
1	2	1	0	19.1066565	75	0
1	2	1	0	20.3017833	75	0
1	2	1	0	17.9849305	50	0
1	2	1	0	21.536	75	0

1	2	1	0	20.6298828	75	0
1	2	1	0	25.4040584	95	1
1	2	1	0	17.1045786	50	0
1	2	1	0	17.1045786	50	0
1	2	1	0	14.6207937	10	0
1	3	2	1	22.6699893	90	1
1	3	2	1	19.9971879	75	0
1	3	2	1	18.5803455	50	0
1	3	2	1	17.1957672	25	0
1	3	2	1	27.086414	95	1
1	3	2	1	22.136954	85	1
1	3	2	1	19.5901689	75	0
1	3	2	1	16.9526627	25	0
1	3	2	1	18.6004162	75	0
1	3	2	1	13.6094675	5	0
1	3	2	1	19.7666365	75	0
1	3	2	1	22.8099129	90	1
1	3	2	1	21.2637741	75	0
1	3	2	1	23.4319527	90	1
1	3	2	1	24.556213	95	1
1	3	2	1	20.1758926	85	1
1	3	2	1	18.1683296	50	0
1	3	2	1	14.5423953	10	0
1	3	2	1	18.5732797	50	0
1	3	2	1	20.3022385	75	0
1	3	2	1	21.3017751	75	0
1	3	2	1	19.8199158	75	0
1	3	2	1	16.184573	10	0
1	3	2	1	17.7934952	25	0
1	3	2	1	16.3001543	10	0
1	3	2	1	16.196955	10	0
1	3	2	1	17.3029526	10	0
1	3	2	1	17.6923077	10	0
1	3	2	1	21.4274623	75	0
1	3	2	1	20.2140309	75	0
1	3	2	1	15.8730159	10	0
1	3	2	1	24.1088867	85	1
1	3	2	1	17.7915519	25	0
1	3	2	1	19.7057175	50	0
1	3	2	1	19.9437557	50	0
1	3	2	1	21.0059172	75	0
1	3	2	1	17.4814987	10	0
1	3	2	1	19.2263545	50	0
1	3	2	1	17.9075777	25	0
1	3	2	1	23.4565585	75	0

1	3	2	1	21.7450286	50	0
1	3	2	1	25.854109	75	0
1	3	2	1	27.8784791	85	1
1	3	2	1	18.3784919	25	0
1	3	2	1	23.8048006	75	0
1	3	2	1	17.4202815	10	0
1	3	2	1	21.0566653	75	0
1	3	2	1	22.6835832	75	0
1	3	2	1	22.7040816	50	0
1	3	2	1	23.556834	50	0
1	3	2	1	23.2928241	50	0
1	3	2	1	20.608	50	0
1	3	2	1	23.8821019	75	0
1	3	2	1	17.5561711	10	0
1	3	2	1	21.1248285	50	0
1	3	2	1	21.1248285	50	0
1	3	2	1	19.5266272	25	0
1	3	2	1	16.9859657	10	0
1	3	2	1	20.6811541	50	0
1	3	2	1	32.5368169	95	1
1	3	2	1	26.611797	85	1
1	3	2	1	24.0640474	75	0
1	3	2	1	24.14119	75	0
1	3	2	1	27.2242638	85	1
1	3	2	1	25.1239669	50	0
1	3	2	1	25.274859	50	0
1	3	2	1	24.5949074	50	0
1	3	2	1	18.9998123	10	0
1	3	2	1	26.4625608	75	0
1	3	2	1	14.8412148	5	0
1	3	2	1	25.7887517	85	1
1	3	2	1	22.1685343	25	0
1	3	2	1	24.7654566	50	0
1	3	2	1	19.5398101	10	0
1	3	2	1	21.9706981	25	0
1	3	2	1	20.3332672	25	0
1	3	2	1	27.8067398	75	0
1	3	2	1	25.7690542	75	0
1	3	2	1	30.4271979	90	1
1	3	2	1	20.661157	25	0
1	3	2	1	21.7360285	25	0
1	3	2	1	24.8601466	75	0
1	3	2	1	29.6158997	90	1
1	3	2	1	18.2613266	10	0
1	3	2	1	24.0365105	50	0

1	3	2	1	32.64	95	1
1	3	2	1	27.1203156	75	0
1	3	2	1	27.9191439	90	1
1	3	2	1	17.6354627	5	0
1	3	2	1	24.9232936	75	0
1	3	2	1	20.531401	25	0
1	3	2	1	24.4699938	75	0
1	3	2	1	20.3209094	25	0
1	3	2	1	24.8702422	75	0
1	3	2	1	22.984009	50	0
1	3	2	1	19.5517733	25	0
1	3	2	1	20.8490519	50	0
1	3	2	1	24.9323621	75	0
1	3	2	1	24.3040486	75	0
1	3	2	1	26.228191	75	0
1	3	2	1	24.8941858	50	0
1	3	2	1	23.8913816	50	0
1	3	2	1	19.2343779	10	0
1	3	2	1	22.1753299	50	0
1	3	2	1	22.7635715	50	0
1	3	2	1	29.7560008	85	1
1	3	2	1	26.8929396	75	0
1	3	2	1	22.8129339	50	0
1	3	2	1	27.3979303	75	0
1	3	2	1	32.2195244	95	1
1	3	2	1	18.7652468	10	0
1	3	2	1	21.1218837	50	0
1	3	2	1	20.501097	25	0
1	3	2	1	27.8742284	90	1

nutrition	waist	waist/length index	central obesity	CT	TG	LDLC	HDLC
1				96	64	54	30
1				178	99	110	49
1				144	67	91	39
1				180	88	116	46
1				184	111	108	54
1				114	50	88	16
1				149	76	103	31
1				146	92	98	30
1	57	0.75	1	201	70	149	38
1				160	95	84	57
1	47	0.580246914	1	196	101	137	39
1	48	0.568047337	1	100	53	60	30
1				154	57	95	48
1				169	120	105	40
0				129	217	61	25
2				180	50	110	60
1				182	68	126	43
1				182	86	124	41
1				180	84	116	48
1				118	60	77	29
0				115	85	60	38
1				150	115	89	39
1	42	0.512195122	1	218	53	145	62
1				211	107	146	44
1				146	46	82	55
3				96	63	58	26
1				138	82	85	37
1				104	87	59	28
1				142	76	97	30
1				117	126	64	28
1				172	97	103	50
1				179	116	129	27
3				137	67	86	38
0	41	0.482352941	0	103	119	59	21
1				101	122	59	18
1	43	0.507674144	1	186	145	126	31
1				163	58	111	41
1				184	115	118	43
1				141	79	96	29
1				133	81	97	20
1				109	89	59	32
1				192	93	137	36
1	47	0.494736842	0	120	41	65	47

1	44	0.523809524	1	164	63	114	37
2	57	0.647727273	1	142	70	94	34
1	45.5	0.505555556	1	138	74	81	42
1				165	114	33	33
1				182	71	112	56
2				136	76	77	44
1				146	66	96	37
2				175	91	122	34
0				197	126	135	37
1	47	0.544611819	1	218	80	146	56
1				195	101	116	58
1				171	53	123	37
1				169	42	124	37
1				169	42	124	37
1	52	0.587570621	1	161	80	112	32
1				131	135	69	35
1				172	87	115	40
1	47	0.5875	1	140	54	80	50
1	51	0.6	1	147	74	98	34
1				145	74	97	33
1				249	206	171	37
1	51	0.6	1	148	64	102	33
1				142	119	84	35
1				157	69	103	41
1				200	69	149	37
1	45	0.529411765	1	110	68	69	28
1				158	102	97	40
1	49	0.371212121	0	117	122	43	50
1				198	83	140	42
1				190	160	126	32
1				171	120	106	41
1	53	0.602272727	1	127	91	78	31
1				172	53	112	49
0				149	38	87	55
1				129	63	80	36
3	63	0.611650485	1	170	122	122	24
3				117	66	68	36
2	56	0.647398844	1	192	65	131	48
1	49.5	0.532258065	1	205	138	132	45
1				118	53	78	29
1	45	0.505050505	1	182	159	114	37
1				109	85	68	24
1				176	59	113	51
0	41	0.422680412	0	162	79	100	47
2				150	83	94	40

2				152	128	89	38
1	46	0.319444444	0	183	51	136	37
1				182	100	117	45
1				161	32	78	77
2	66	0.647058824	1	188	89	135	36
3				214	92	153	43
1				116	67	72	30
1				189	59	120	57
0				142	117	85	34
1				163	82	102	45
1				139	79	86	37
1				167	46	121	37
1				194	161	128	34
3	59	0.437037037	0	118	55	57	50
2				194	93	132	44
1				166	61	110	44
1				153	66	90	50
1				148	105	94	33
2				186	52	126	49
1				157	79	89	52
1	51.5	0.572222222	1	121	81	76	28
3				192	122	131	37
1				133	113	71	40
3				171	53	108	52
1				182	117	121	38
1				206	132	141	39
0				154	48	100	44
1				162	64	111	38
1	53	0.552083333	1	101	68	57	30
1				135	79	76	44
0				168	99	115	34
1	51	0.536842105	1	150	41	97	45
1				136	65	93	30
1				146	71	85	46
1				156	74	100	41
2				176	97	111	46
1	51.5	0.530927835	1	128	33	61	61
1	50	0.549450549	1	139	116	86	30
1				154	96	82	53
1				162	74	94	53
1				179	129	119	34
2				135	63	85	37
1	54	0.495412844	0	152	81	99	37
1				200	63	127	60
1				145	81	86	43

1	50	0.520833333	1	184	84	117	50
1	48	0.436363636	0	165	87	106	42
1	51	0.504950495	1	167	97	102	45
1				196	70	126	56
1	53.5	0.469298246	0	103	58	72	19
1				138	25	73	60
1				152	37	94	51
3				154	53	104	40
0				164	106	117	26
1				158	126	101	32
1	51	0.541401274	1	222	110	143	57
1				157	59	96	50
1	54	0.52173913	1	171	82	97	58
2				180	365	73	34
1	51	0.542553191	1	161	33	111	43
1				153	55	100	42
1	57.5	0.508849558	1	122	62	82	27
1				128	70	83	31
1				179	90	117	44
1	59	0.551401869	1	122	62	82	27
1	47	0.443396226	0	154	131	90	38
1	50	0.471698113	0	146	37	95	44
1				181	43	132	40
1				127	81	85	26
2	60	0.582524272	1	152	36	97	48
1	49	0.556818182	1	129	45	71	49
1				129	49	78	41
2				161	70	100	47
3	66.5	0.536290323	1	178	108	84	72
1				204	63	126	65
1				135	65	86	36
1				160	78	96	49
1	46	0.429906542	0	138	92	76	43
1				178	115	118	37
1				118	55	73	34
1				138	58	71	56
1				167	117	96	48
1				176	34	108	62
1				184	134	118	39
1	57	0.368693402	0	164	79	109	40
1				179	91	104	57
1				132	115	81	28
1	51	0.64556962	1	195	52	135	50
1				206	84	140	50
0	50	0.472143532	0	111	55	68	33

1	56.2	0.534220532	1	176	63	108	56
1				189	41	103	78
1	59	0.50212766	1	145	31	88	51
2				142	66	89	39
1				165	71	103	48
2				173	98	101	53
2				174	80	100	58
1				169	47	97	63
1				131	69	79	38
2				185	69	125	46
1				166	71	94	58
1				164	120	101	40
2				146	68	91	41
1				177	156	107	39
1				166	42	104	53
1				141	43	101	32
1	52	0.581005587	1	123	58	63	49
1	54	0.438311688	0	143	73	85	43
1				176	58	107	57
1	46	0.431924883	0	191	70	124	53
1				177	72	130	33
0				158	87	98	43
3				161	53	113	37
1				163	55	98	54
1	56.5	0.538095238	1	180	44	110	62
1	53	0.481818182	0	177	93	95	63
1				165	54	107	48
1				157	81	92	49
2				153	79	92	46
1				167	46	107	51
2				144	74	83	46
0				161	76	92	54
0				160	120	103	33
1	52	0.462222222	0	161	59	90	60
1	51	0.466179159	0	135	104	76	38
0	55	0.555555556	1	202	140	113	61
1	60	0.508474576	1	195	127	132	38
1				185	136	124	34
1				116	43	71	36
1				141	94	78	44
2	62	0.492063492	0	217	87	163	36
1	57	0.401408451	0	116	27	63	47
1				201	119	115	62
0				142	91	74	50
1				128	66	75	40

1				175	70	120	41
1				112	46	66	37
1				152	85	96	39
1				120	85	70	33
1				156	85	94	45
1	51	0.447368421	0	185	120	116	45
1				154	154	91	32
1	62	0.556053812	1	176	83	119	41
1				147	77	78	54
1	63.5	0.5	1	160	78	103	42
1				143	116	83	36
1				170	80	113	41
1	59.5	0.5	1	144	87	80	47
2				154	115	98	33
1				171	48	106	56
1	67	0.531746032	1	223	126	151	47
1				131	66	69	49
1				144	49	86	48
1	63	0.518518519	1	127	54	73	43
1				140	118	83	33
1				128	100	72	36
1				155	99	96	40
0	52	0.597701149	1	209	138	138	43
1				127	54	67	50
2	72	0.566929134	1	150	61	78	60
1				129	90	66	45
1				168	102	80	68
1	60.5	0.441605839	0	138	85	79	42
1				130	48	81	39
1				128	74	62	51
2	79	0.593984962	1	171	99	114	38
3				109	74	54	40
1	56	0.504504505	1	153	184	80	37
1				144	117	90	31
1				142	55	89	42
1				115	46	61	45
1	66.5	0.573275862	1	137	85	82	38
1	61	0.5	1	139	59	92	36
1	75.2	0.572298326	1	162	108	98	42
1				85	135	29	29
1	68	0.498168498	0	186	165	108	45
1				135	60	84	39
1				172	58	107	53
1				139	124	72	42
1	67	0.817073171	1	172	127	106	41

3	84	0.633962264	1	171	131	82	63
1				167	93	69	80
1				167	93	69	80
1	54	0.437246964	0	194	168	116	44
2				163	91	101	44
1				164	54	109	44
1				117	59	70	35
1	61.5	0.804973822	1	96	79	55	26
3				173	156	116	26
2				161	93	98	45
1				168	182	99	33
1	64	0.492307692	0	173	60	129	31
1	65	0.481481481	0	173	78	113	45
0	57	0.438461538	0	150	74	87	48
1	67	0.489051095	0	172	104	106	45
2				200	148	123	47
1	74	0.560606061	1	149	132	84	39
2	71	0.546153846	1	157	160	91	34
3	71	0.546153846	1	168	72	111	43
2	68.5	0.479356193	0	174	98	121	33
1	63.5	0.494163424	0	114	49	62	42
1				154	92	100	36
1				181	59	115	54
1				144	87	92	35
1	67	0.515384615	1	190	158	110	48
1				153	67	98	42
1	57	0.431818182	0	156	47	95	51
1				150	52	100	40
1	66	0.458333333	0	159	73	108	37
1	58.5	0.397959184	0	137	64	84	40
1				126	84	78	31
1				88	126	37	26
1				161	100	94	46
1				159	148	93	37
1				176	56	117	48
2	80.5	0.609848485	1	163	262	78	33
1	61	0.638743455	1	182	153	120	31
1				152	75	94	43
1	68	0.53125	1	181	183	103	41
1	71.5	0.55	1	181	118	113	45
1				160	59	103	45
1				134	87	73	44
1				163	84	104	42
1				138	98	96	23
1				168	210	90	36

1	86	0.603508772	1	216	314	123	30
2				139	250	52	38
1				126	67	84	29
1				161	93	98	44
1				117	57	73	32
1	68	0.474198047	0	136	52	76	49
1	79	0.516339869	1	131	185	59	35
1	88	0.628571429	1	116	46	60	47
1				147	100	79	48
1	85	0.590277778	1	142	100	78	44
1	70	0.714285714	1	174	210	90	42
1	78	0.546218487	1	158	90	104	36
1				133	91	72	43
1	71	0.525925926	1	173	117	107	43
1	71	0.696078431	1	173	117	107	43
1	70	0.538461538	1	225	298	132	33
1	61	0.64893617	1	127	62	86	29
1	76	0.513513514	1	196	177	118	43
3	103	0.656050955	1	144	150	87	27
2	78	0.577777778	1	93	70	48	32
1				182	83	117	49
1	85	0.62962963	1	193	105	130	42
2	81.5	0.586330935	1	163	74	98	50
1	78	1.018276762	1	145	109	92	32
1				128	63	75	40
1	84	0.626865672	1	146	92	87	41
1				118	126	64	29
1	95	0.597484277	1	176	383	75	24
0				172	116	89	60
2				122	114	67	32
1				160	103	99	41
1	81	0.618320611	1	151	36	101	43
1				141	67	97	31
1				135	152	67	38
1				134	93	69	46
1				182	107	119	42
1				241	271	156	31
2	89	0.566878981	1	146	71	98	33
1	75	0.669642857	1	151	94	100	32
1				190	103	123	47
1				144	94	105	20
2	97.5	0.636007828	1	199	285	111	32
1				164	121	92	48
1				130	122	73	33
3	76	0.487179487	0	168	159	92	43

1	91	0.758965805	1	166	144	98	39
2	90.5	0.786956522	1	143	157	72	40
0				120	97	63	38
1				164	130	102	36
1				150	282	63	31
1	78	0.548137737	1	156	74	91	51
1				132	105	75	36
1	73	0.536764706	1	166	121	105	37
1				190	128	128	36
1	67	0.451786918	0	164	121	92	48
1				147	122	83	40
1				148	156	84	33
1				211	130	127	58
1				204	176	121	47
1				100	44	49	43
1				152	58	94	47
1				169	64	116	40
1				136	76	70	51
1	75.5	0.535460993	1	121	146	66	26
2				170	72	99	56
1				174	80	114	44
1				177	84	115	45
1				180	84	120	44
3				224	111	143	59
1				136	60	86	38
1				164	67	108	42
1	75	0.646551724	1	162	105	99	42
2				144	120	89	32

non-hdl chol	high chol	chol classific	high LDL	LDL classific	high TG	TG classific	low HDL
66.4	0	0	0	0	0	0	1
129.5	0	1	0	0	0	1	0
104.8	0	0	0	0	0	0	1
134	0	1	0	1	0	0	0
130.1	0	1	0	0	1	2	0
98.4	0	0	0	0	0	0	1
117.7	0	0	0	0	0	1	1
116.4	0	0	0	0	0	1	1
162.6	1	2	1	2	0	0	1
102.6	0	0	0	0	0	1	0
156.8	0	1	1	2	1	2	1
70.2	0	0	0	0	0	0	1
106	0	0	0	0	0	0	0
128.9	0	0	0	0	1	2	0
104.5	0	0	0	0	1	2	1
120.2	0	1	0	1	0	0	0
139.5	0	1	0	1	0	0	0
141.4	0	1	0	1	0	1	0
132.4	0	1	0	1	0	1	0
89.3	0	0	0	0	0	0	1
77.1	0	0	0	0	0	1	1
111.5	0	0	0	0	1	2	1
156	1	2	1	2	0	0	0
167.5	1	2	1	2	1	2	0
91	0	0	0	0	0	0	0
70.2	0	0	0	0	0	0	1
101.2	0	0	0	0	0	1	1
76.3	0	0	0	0	0	1	1
111.9	0	0	0	0	0	1	1
89	0	0	0	0	1	2	1
122	0	1	0	0	0	1	0
152.2	0	1	0	1	1	2	1
99.1	0	0	0	0	0	0	1
82.4	0	0	0	0	1	2	1
83.5	0	0	0	0	1	2	1
155.4	0	1	0	1	1	2	1
122.4	0	0	0	1	0	0	0
140.7	0	1	0	1	1	2	0
111.7	0	0	0	0	0	1	1
112.8	0	0	0	0	0	1	1
77.2	0	0	0	0	0	1	1
156	0	1	1	2	0	1	1
72.8	0	0	0	0	0	0	0

126.6	0	0	0	1	0	0	1
108	0	0	0	0	0	0	1
95.9	0	0	0	0	0	0	0
132.3	0	0	0	0	1	2	1
125.8	0	1	0	1	0	0	0
92.2	0	0	0	0	0	1	0
109.2	0	0	0	0	0	0	1
140.6	0	1	0	1	0	1	1
160.5	0	1	1	2	1	2	1
162.3	1	2	1	2	0	1	0
136.6	0	1	0	1	1	2	0
134	0	1	0	1	0	0	1
132.1	0	0	0	1	0	0	1
132.1	0	0	0	1	0	0	1
129	0	0	0	1	0	1	1
96	0	0	0	0	1	2	1
132.3	0	1	0	1	0	1	1
90.4	0	0	0	0	0	0	0
113.2	0	0	0	0	0	0	1
111.8	0	0	0	0	0	0	1
212.4	1	2	1	2	1	2	1
114.7	0	0	0	0	0	0	1
107.3	0	0	0	0	1	2	1
116.5	0	0	0	0	0	0	0
163.2	1	2	1	2	0	0	1
82.4	0	0	0	0	0	0	1
117.8	0	0	0	0	1	2	0
67.3	0	0	0	0	1	2	0
156.1	0	1	1	2	0	1	0
157.7	0	1	0	1	1	2	1
130.1	0	1	0	0	1	2	0
96	0	0	0	0	0	1	1
123	0	1	0	1	0	0	0
94.4	0	0	0	0	0	0	0
93	0	0	0	0	0	0	1
146.4	0	1	0	1	1	2	1
81.2	0	0	0	0	0	0	1
144.2	0	1	1	2	0	0	0
159.9	1	2	1	2	1	2	0
88.8	0	0	0	0	0	0	1
145.5	0	1	0	1	1	2	1
84.9	0	0	0	0	0	1	1
124.8	0	1	0	1	0	0	0
115.5	0	0	0	0	0	1	0
110.4	0	0	0	0	0	1	1

114.1	0	0	0	0	1	2	1
146.1	0	1	1	2	0	0	1
137.2	0	1	0	1	1	2	0
84.4	0	0	0	0	0	0	0
152.3	0	1	1	2	0	1	1
171.4	1	2	1	2	0	1	0
85.7	0	0	0	0	0	0	1
131.7	0	1	0	1	0	0	0
108.4	0	0	0	0	1	2	1
117.9	0	0	0	0	0	1	0
101.7	0	0	0	0	0	1	1
129.9	0	0	0	1	0	0	1
159.7	0	1	0	1	1	2	1
67.8	0	0	0	0	0	0	0
150.3	0	1	1	2	0	1	0
122.4	0	0	0	1	0	0	0
103.4	0	0	0	0	0	0	0
115.3	0	0	0	0	1	2	1
136.6	0	1	0	1	0	0	0
104.7	0	0	0	0	0	1	0
92.6	0	0	0	0	0	1	1
155.4	0	1	1	2	1	2	1
93.1	0	0	0	0	1	2	1
118.8	0	1	0	0	0	0	0
144.2	0	1	0	1	1	2	1
167.3	1	2	1	2	1	2	1
109.9	0	0	0	0	0	0	0
124.1	0	0	0	1	0	0	1
70.7	0	0	0	0	0	0	1
91.5	0	0	0	0	0	1	0
134.4	0	0	0	1	0	1	1
105.1	0	0	0	0	0	0	0
105.6	0	0	0	0	0	0	1
99.6	0	0	0	0	0	0	0
115	0	0	0	0	0	0	0
129.9	0	1	0	1	0	1	0
67.5	0	0	0	0	0	0	0
109.4	0	0	0	0	1	2	1
101.1	0	0	0	0	0	1	0
109.2	0	0	0	0	0	0	0
145.2	0	1	0	1	1	2	1
97.8	0	0	0	0	0	0	1
115.4	0	0	0	0	0	1	1
139.9	1	2	0	1	0	0	0
101.8	0	0	0	0	0	1	0

134.2	0	1	0	1	0	1	0
123.5	0	0	0	0	0	1	0
121.8	0	0	0	0	0	1	0
139.6	0	1	0	1	0	0	0
83.7	0	0	0	0	0	0	1
78.4	0	0	0	0	0	0	0
101.2	0	0	0	0	0	0	0
114.5	0	0	0	0	0	0	1
138.2	0	0	0	1	1	2	1
126.5	0	0	0	0	1	2	1
165.3	1	2	1	2	1	2	0
107.4	0	0	0	0	0	0	0
112.9	0	1	0	0	0	1	0
146.2	0	1	0	0	1	2	1
117.6	0	0	0	1	0	0	0
110.9	0	0	0	0	0	0	0
94.7	0	0	0	0	0	0	1
97.1	0	0	0	0	0	0	1
134.7	0	1	0	1	0	1	0
94.7	0	0	0	0	0	0	1
116.2	0	0	0	0	1	2	1
102.3	0	0	0	0	0	0	0
140.6	0	1	1	2	0	0	0
101.3	0	0	0	0	0	1	1
104	0	0	0	0	0	0	0
80.2	0	0	0	0	0	0	0
87.9	0	0	0	0	0	0	0
114.3	0	0	0	0	0	0	0
105.7	0	1	0	0	1	2	0
138.6	1	2	0	1	0	0	0
99.3	0	0	0	0	0	0	1
111.1	0	0	0	0	0	1	0
94.7	0	0	0	0	0	1	0
141	0	1	0	1	1	2	1
84	0	0	0	0	0	0	1
82.2	0	0	0	0	0	0	0
119.4	0	0	0	0	1	2	0
114.4	0	1	0	0	0	0	0
144.6	0	1	0	1	1	2	1
124.5	0	0	0	0	0	1	1
122.5	0	1	0	0	0	1	0
104.2	0	0	0	0	1	2	1
144.9	0	1	1	2	0	0	0
156.4	1	2	1	2	0	1	0
78.5	0	0	0	0	0	0	1

120.5	0	1	0	0	0	0	0
110.7	0	1	0	0	0	0	0
94	0	0	0	0	0	0	0
102.6	0	0	0	0	0	0	1
117.2	0	0	0	0	0	0	0
120.3	0	1	0	0	0	1	0
116.1	0	1	0	0	0	1	0
106.4	0	0	0	0	0	0	0
92.7	0	0	0	0	0	0	1
139.1	0	1	0	1	0	0	0
108.5	0	0	0	0	0	0	0
124.5	0	0	0	0	1	2	1
105	0	0	0	0	0	0	0
138.1	0	1	0	0	1	2	1
112.8	0	0	0	0	0	0	0
109.5	0	0	0	0	0	0	1
74.3	0	0	0	0	0	0	0
99.6	0	0	0	0	0	0	0
118.9	0	1	0	0	0	0	0
138.3	0	1	0	1	0	0	0
144.3	0	1	0	1	0	0	1
115	0	0	0	0	0	1	0
123.7	0	0	0	1	0	0	1
109.2	0	0	0	0	0	0	0
118.3	0	1	0	0	0	0	0
113.9	0	1	0	0	0	1	0
117.4	0	0	0	0	0	0	0
107.8	0	0	0	0	0	1	0
107.5	0	0	0	0	0	1	0
115.9	0	0	0	0	0	0	0
97.6	0	0	0	0	0	0	0
107.2	0	0	0	0	0	1	0
126.9	0	0	0	0	1	2	1
101.5	0	0	0	0	0	0	0
96.6	0	0	0	0	1	2	1
140.7	1	2	0	1	1	2	0
157	0	1	1	2	1	2	1
151.5	0	1	0	1	1	2	1
79.7	0	0	0	0	0	0	1
97.1	0	0	0	0	0	1	0
180.8	1	2	1	2	0	1	1
68.6	0	0	0	0	0	0	0
139	1	2	0	1	1	2	0
91.9	0	0	0	0	0	1	0
88	0	0	0	0	0	0	0

133.9	0	1	0	1	0	0	0
75.5	0	0	0	0	0	0	1
112.9	0	0	0	0	0	1	1
86.8	0	0	0	0	0	1	1
111.3	0	0	0	0	0	1	0
140.3	0	1	0	1	1	2	0
122.1	0	0	0	0	1	2	1
135.5	0	1	0	1	0	1	0
93.1	0	0	0	0	0	1	0
118.2	0	0	0	0	0	1	0
106.6	0	0	0	0	1	2	1
128.7	0	1	0	1	0	1	0
97.5	0	0	0	0	0	1	0
120.9	0	0	0	0	1	2	1
115.3	0	1	0	0	0	0	0
176.2	1	2	1	2	1	2	0
82.2	0	0	0	0	0	0	0
96.1	0	0	0	0	0	0	0
84.2	0	0	0	0	0	0	0
106.9	0	0	0	0	1	2	1
92.1	0	0	0	0	1	2	1
115.5	0	0	0	0	0	1	1
166	1	2	1	2	1	2	0
77.5	0	0	0	0	0	0	0
89.8	0	0	0	0	0	0	0
83.9	0	0	0	0	0	1	0
100.5	0	0	0	0	1	2	0
96.1	0	0	0	0	0	1	0
91	0	0	0	0	0	0	1
76.8	0	0	0	0	0	0	0
133.3	0	1	0	1	0	1	1
69.2	0	0	0	0	0	0	1
116.3	0	0	0	0	1	2	1
113.1	0	0	0	0	0	1	1
100.2	0	0	0	0	0	0	0
70	0	0	0	0	0	0	0
99.4	0	0	0	0	0	0	1
103.4	0	0	0	0	0	0	1
119.9	0	0	0	0	0	1	0
55.8	0	0	0	0	1	2	1
141.2	0	1	0	0	1	2	0
96.4	0	0	0	0	0	0	1
118.7	0	1	0	0	0	0	0
97.2	0	0	0	0	0	1	0
131	0	1	0	0	1	2	0

107.7	0	1	0	0	1	2	0
87.2	0	0	0	0	0	1	0
87.2	0	0	0	0	0	1	0
150	0	1	0	1	1	2	0
118.7	0	0	0	0	0	1	0
120.2	0	0	0	0	0	0	0
82.1	0	0	0	0	0	0	1
70.3	0	0	0	0	0	0	1
146.7	0	1	0	1	1	2	1
116.3	0	0	0	0	0	1	0
135	0	0	0	0	1	2	1
141.8	0	1	0	1	0	0	1
128.3	0	1	0	1	0	0	0
102.2	0	0	0	0	0	0	0
127	0	1	0	0	0	1	0
152.7	1	2	0	1	1	2	0
110	0	0	0	0	1	2	1
123.4	0	0	0	0	1	2	1
125	0	0	0	1	0	0	0
140.7	0	1	0	1	0	1	1
72.1	0	0	0	0	0	0	0
117.9	0	0	0	0	0	1	1
127.2	0	1	0	1	0	0	0
109.4	0	0	0	0	0	0	1
142	0	1	0	1	1	2	0
111.3	0	0	0	0	0	0	0
104.8	0	0	0	0	0	0	0
110	0	0	0	0	0	0	0
122.4	0	0	0	0	0	0	1
96.8	0	0	0	0	0	0	0
95.2	0	0	0	0	0	0	1
61.7	0	0	0	0	0	1	1
115	0	0	0	0	0	1	0
122.5	0	0	0	0	1	2	1
127.9	0	1	0	1	0	0	0
130	0	0	0	0	1	2	1
150.8	0	1	0	1	1	2	1
109.2	0	0	0	0	0	0	0
139.7	0	1	0	0	1	2	0
136.4	0	1	0	1	0	1	0
114.6	0	0	0	0	0	0	0
89.9	0	0	0	0	0	0	0
121	0	0	0	0	0	0	0
115.5	0	0	0	0	0	1	1
132	0	0	0	0	1	2	1

186.1	1	2	0	1	1	2	1
101.5	0	0	0	0	1	2	1
97.3	0	0	0	0	0	0	1
117	0	0	0	0	0	1	0
84.6	0	0	0	0	0	0	1
86.6	0	0	0	0	0	0	0
96.2	0	0	0	0	1	2	1
68.9	0	0	0	0	0	0	0
99.3	0	0	0	0	0	1	0
97.7	0	0	0	0	0	1	0
132.3	0	1	0	0	1	2	0
121.8	0	0	0	0	0	1	1
90.2	0	0	0	0	0	1	0
130.2	0	1	0	0	0	1	0
130.2	0	1	0	0	0	1	0
191.7	1	2	1	2	1	2	1
98.2	0	0	0	0	0	0	1
153.3	0	1	0	1	1	2	0
116.9	0	0	0	0	1	2	1
61.5	0	0	0	0	0	0	1
133.4	0	1	0	1	0	0	0
151.1	0	1	1	2	0	1	0
113	0	0	0	0	0	0	0
113.4	0	0	0	0	0	1	1
88	0	0	0	0	0	0	0
105.2	0	0	0	0	0	1	0
89	0	0	0	0	0	1	1
151.8	0	1	0	0	1	2	1
111.9	0	1	0	0	0	1	0
89.8	0	0	0	0	1	2	1
119.5	0	0	0	0	0	1	0
108	0	0	0	0	0	0	0
110.4	0	0	0	0	0	0	1
97.3	0	0	0	0	1	2	1
87.6	0	0	0	0	0	1	0
140	0	1	0	1	0	1	0
209.8	1	2	1	2	1	2	1
112.6	0	0	0	0	0	0	1
119.1	0	0	0	0	0	1	1
143.4	0	1	0	1	0	1	0
124.1	0	0	0	0	0	1	1
167.5	0	1	0	1	1	2	1
115.9	0	0	0	0	0	1	0
97.3	0	0	0	0	0	1	1
125	0	0	0	0	1	2	0

126.9	0	0	0	0	1	2	1
102.9	0	0	0	0	1	2	0
82	0	0	0	0	0	1	1
127.7	0	0	0	0	1	2	1
119.2	0	0	0	0	1	2	1
105.4	0	0	0	0	0	0	0
96.2	0	0	0	0	0	1	1
128.7	0	0	0	0	0	1	1
154	0	1	0	1	0	1	1
115.9	0	0	0	0	0	1	0
107.1	0	0	0	0	0	1	1
115.5	0	0	0	0	1	2	1
153.2	1	2	0	1	1	2	0
156.6	1	2	0	1	1	2	0
57.4	0	0	0	0	0	0	0
105.4	0	0	0	0	0	0	0
129.2	0	0	0	1	0	0	1
85.2	0	0	0	0	0	0	0
94.8	0	0	0	0	1	2	1
113.7	0	1	0	0	0	0	0
129.6	0	1	0	1	0	0	0
131.7	0	1	0	1	0	0	0
136.3	0	1	0	1	0	0	0
164.8	1	2	1	2	0	1	0
97.6	0	0	0	0	0	0	1
121.6	0	0	0	0	0	0	0
120.2	0	0	0	0	0	1	0
112.5	0	0	0	0	0	1	1

HDL classific	high non-HDL	non-HDL classific	dyslipidemia classification	high chol and low HDL	muliple dyslip	dysl
2	0	0	3	0	0	
0	0	1	0	0	0	
2	0	0	3	0	0	
0	0	1	0	0	0	
0	0	1	2	0	0	
2	0	0	3	0	0	
2	0	0	3	0	0	
2	0	0	3	0	0	
2	1	2	4	1	0	
0	0	0	0	0	0	
2	1	2	4	0	1	
2	0	0	3	0	0	
0	0	0	0	0	0	
1	0	1	2	0	0	
2	0	0	4	0	0	
0	0	1	0	0	0	
1	0	1	0	0	0	
1	0	1	0	0	0	
0	0	1	0	0	0	
2	0	0	3	0	0	
2	0	0	3	0	0	
2	0	0	4	0	0	
0	1	2	1	0	0	
1	1	2	4	0	0	
0	0	0	0	0	0	
2	0	0	3	0	0	
2	0	0	3	0	0	
2	0	0	3	0	0	
2	0	0	3	0	0	
2	0	0	4	0	0	
0	0	1	0	0	0	
2	1	2	4	0	0	
2	0	0	3	0	0	
2	0	0	4	0	0	
2	0	0	4	0	0	
2	1	2	4	0	0	
1	0	1	0	0	0	
1	0	1	2	0	0	
2	0	0	3	0	0	
2	0	0	3	0	0	
2	0	0	3	0	0	
2	1	2	4	1	0	
0	0	0	0	0	0	

2	0	1	3	0	0
2	0	0	3	0	0
1	0	0	0	0	0
2	0	1	4	0	0
0	0	1	0	0	0
1	0	0	0	0	0
2	0	0	3	0	0
2	0	1	3	0	0
2	1	2	4	0	1
0	1	2	1	0	0
0	0	1	2	0	0
2	0	1	3	0	0
2	0	1	3	0	0
2	0	1	3	0	0
2	0	1	3	0	0
2	0	0	4	0	0
2	0	1	3	0	0
0	0	0	0	0	0
2	0	0	3	0	0
2	0	0	3	0	0
2	1	2	4	0	1
2	0	0	3	0	0
2	0	0	4	0	0
1	0	0	0	0	0
2	1	2	4	1	0
2	0	0	3	0	0
1	0	0	2	0	0
0	0	0	2	0	0
1	1	2	1	0	0
2	1	2	4	0	0
1	0	1	2	0	0
2	0	0	3	0	0
0	0	1	0	0	0
0	0	0	0	0	0
2	0	0	3	0	0
2	1	2	4	0	0
2	0	0	3	0	0
0	0	1	1	0	0
0	1	2	4	0	0
2	0	0	3	0	0
2	1	2	4	0	0
2	0	0	3	0	0
0	0	1	0	0	0
0	0	0	0	0	0
2	0	0	3	0	0

2	0	0	4	0	0
2	1	2	4	1	0
1	0	1	2	0	0
0	0	0	0	0	0
2	1	2	4	1	0
1	1	2	1	0	0
2	0	0	3	0	0
0	0	1	0	0	0
2	0	0	4	0	0
0	0	0	0	0	0
2	0	0	3	0	0
2	0	1	3	0	0
2	1	2	4	0	0
0	0	0	0	0	0
1	1	2	1	0	0
1	0	1	0	0	0
0	0	0	0	0	0
2	0	0	4	0	0
0	0	1	0	0	0
0	0	0	0	0	0
2	0	0	3	0	0
2	1	2	4	0	1
2	0	0	4	0	0
0	0	0	0	0	0
2	0	1	4	0	0
2	1	2	4	0	1
1	0	0	0	0	0
2	0	1	3	0	0
2	0	0	3	0	0
1	0	0	0	0	0
2	0	1	3	0	0
1	0	0	0	0	0
2	0	0	3	0	0
0	0	0	0	0	0
1	0	0	0	0	0
0	0	1	0	0	0
0	0	0	0	0	0
2	0	0	4	0	0
0	0	0	0	0	0
0	0	0	0	0	0
2	1	2	4	0	0
2	0	0	3	0	0
2	0	0	3	0	0
0	0	1	1	0	0
1	0	0	0	0	0

0	0	1	0	0	0
1	0	1	0	0	0
0	0	1	0	0	0
0	0	1	0	0	0
2	0	0	3	0	0
0	0	0	0	0	0
0	0	0	0	0	0
2	0	0	3	0	0
2	0	1	4	0	0
2	0	1	4	0	0
0	1	2	4	0	0
0	0	0	0	0	0
0	0	0	0	0	0
2	1	2	4	0	0
1	0	0	0	0	0
1	0	0	0	0	0
2	0	0	3	0	0
2	0	0	3	0	0
1	0	1	0	0	0
2	0	0	3	0	0
2	0	0	4	0	0
1	0	0	0	0	0
1	0	1	1	0	0
2	0	0	3	0	0
0	0	0	0	0	0
0	0	0	0	0	0
1	0	0	0	0	0
0	0	0	0	0	0
0	0	0	2	0	0
0	0	1	1	0	0
2	0	0	3	0	0
0	0	0	0	0	0
1	0	0	0	0	0
2	0	1	4	0	0
2	0	0	3	0	0
0	0	0	0	0	0
0	0	0	2	0	0
0	0	0	0	0	0
2	0	1	4	0	0
2	0	1	3	0	0
0	0	1	0	0	0
2	0	0	4	0	0
0	0	1	1	0	0
0	1	2	1	0	0
2	0	0	3	0	0

0	0	1	0	0	0
0	0	0	0	0	0
0	0	0	0	0	0
2	0	0	3	0	0
0	0	0	0	0	0
0	0	1	0	0	0
0	0	0	0	0	0
0	0	0	0	0	0
2	0	0	3	0	0
0	0	1	0	0	0
0	0	0	0	0	0
2	0	1	4	0	0
1	0	0	0	0	0
2	0	1	4	0	0
0	0	0	0	0	0
2	0	0	3	0	0
0	0	0	0	0	0
1	0	0	0	0	0
0	0	0	0	0	0
0	0	1	0	0	0
2	0	1	3	0	0
1	0	0	0	0	0
2	0	1	3	0	0
0	0	0	0	0	0
0	0	0	0	0	0
0	0	0	0	0	0
0	0	0	0	0	0
0	0	0	0	0	0
0	0	0	0	0	0
0	0	0	0	0	0
0	0	0	0	0	0
0	0	0	0	0	0
2	0	1	4	0	0
0	0	0	0	0	0
2	0	0	4	0	0
0	0	1	4	0	0
2	1	2	4	0	1
2	1	2	4	0	0
2	0	0	3	0	0
1	0	0	0	0	0
2	1	2	4	1	0
0	0	0	0	0	0
0	0	1	4	0	0
0	0	0	0	0	0
1	0	0	0	0	0

1	0	1	0	0	0
2	0	0	3	0	0
2	0	0	3	0	0
2	0	0	3	0	0
1	0	0	0	0	0
1	0	1	2	0	0
2	0	1	4	0	0
1	0	1	0	0	0
0	0	0	0	0	0
1	0	0	0	0	0
2	0	0	4	0	0
1	0	1	0	0	0
0	0	0	0	0	0
2	0	1	4	0	0
0	0	0	0	0	0
0	1	2	4	0	0
0	0	0	0	0	0
0	0	0	0	0	0
1	0	0	0	0	0
2	0	0	4	0	0
2	0	0	4	0	0
2	0	0	3	0	0
1	1	2	4	0	0
0	0	0	0	0	0
0	0	0	0	0	0
0	0	0	0	0	0
0	0	0	2	0	0
1	0	0	0	0	0
2	0	0	3	0	0
0	0	0	0	0	0
2	0	1	3	0	0
2	0	0	3	0	0
2	0	0	4	0	0
2	0	0	3	0	0
1	0	0	0	0	0
1	0	0	0	0	0
2	0	0	3	0	0
2	0	0	3	0	0
1	0	0	0	0	0
2	0	0	4	0	0
1	0	1	2	0	0
2	0	0	3	0	0
0	0	0	0	0	0
1	0	0	0	0	0
1	0	1	2	0	0

0	0	0	2	0	0
0	0	0	0	0	0
0	0	0	0	0	0
1	1	2	2	0	0
1	0	0	0	0	0
1	0	1	0	0	0
2	0	0	3	0	0
2	0	0	3	0	0
2	1	2	4	0	0
1	0	0	0	0	0
2	0	1	4	0	0
2	0	1	3	0	0
1	0	1	0	0	0
0	0	0	0	0	0
1	0	1	0	0	0
0	1	2	4	0	0
2	0	0	4	0	0
2	0	1	4	0	0
1	0	1	0	0	0
2	0	1	3	0	0
1	0	0	0	0	0
2	0	0	3	0	0
0	0	1	0	0	0
2	0	0	3	0	0
0	0	1	2	0	0
1	0	0	0	0	0
0	0	0	0	0	0
1	0	0	0	0	0
2	0	1	3	0	0
1	0	0	0	0	0
2	0	0	3	0	0
2	0	0	3	0	0
0	0	0	0	0	0
2	0	1	4	0	0
0	0	1	0	0	0
2	0	1	4	0	0
2	1	2	4	0	0
1	0	0	0	0	0
1	0	1	2	0	0
1	0	1	0	0	0
0	0	0	0	0	0
1	0	0	0	0	0
1	0	1	0	0	0
2	0	0	3	0	0
2	0	1	4	0	0

2	1	2	4	0	1
2	0	0	4	0	0
2	0	0	3	0	0
1	0	0	0	0	0
2	0	0	3	0	0
0	0	0	0	0	0
2	0	0	4	0	0
0	0	0	0	0	0
0	0	0	0	0	0
1	0	0	0	0	0
1	0	1	2	0	0
2	0	1	3	0	0
1	0	0	0	0	0
1	0	1	0	0	0
1	0	1	0	0	0
2	1	2	4	0	1
2	0	0	3	0	0
1	1	2	2	0	0
2	0	0	4	0	0
2	0	0	3	0	0
0	0	1	0	0	0
1	1	2	1	0	0
0	0	0	0	0	0
2	0	0	3	0	0
1	0	0	0	0	0
1	0	0	0	0	0
2	0	0	3	0	0
2	1	2	4	0	0
0	0	0	0	0	0
2	0	0	4	0	0
1	0	0	0	0	0
1	0	0	0	0	0
2	0	0	3	0	0
2	0	0	4	0	0
0	0	0	0	0	0
1	0	1	0	0	0
2	1	2	4	0	1
2	0	0	3	0	0
2	0	0	3	0	0
0	0	1	0	0	0
2	0	1	3	0	0
2	1	2	4	0	0
0	0	0	0	0	0
2	0	0	3	0	0
1	0	1	2	0	0

2	0	1	4	0	0
1	0	0	2	0	0
2	0	0	3	0	0
2	0	1	4	0	0
2	0	0	4	0	0
0	0	0	0	0	0
2	0	0	3	0	0
2	0	1	3	0	0
2	1	2	3	0	0
0	0	0	0	0	0
2	0	0	3	0	0
2	0	0	4	0	0
0	1	2	4	0	0
0	1	2	4	0	0
1	0	0	0	0	0
0	0	0	0	0	0
2	0	1	3	0	0
0	0	0	0	0	0
2	0	0	4	0	0
0	0	0	0	0	0
1	0	1	0	0	0
0	0	1	0	0	0
1	0	1	0	0	0
0	1	2	1	0	0
2	0	0	3	0	0
1	0	1	0	0	0
1	0	1	0	0	0
2	0	0	3	0	0

dyslipidemia	simple classif	atherogenic dyslipidemia	mixed dyslipidemia	border or highchol	border or high triglyd
1	1	0	0	0	
0	0	0	0	1	
1	1	0	0	0	
0	0	0	0	1	
1	1	0	0	1	
1	1	0	0	0	
1	1	0	0	0	
1	1	0	0	0	
1	2	0	0	1	
0	0	0	0	0	
1	2	1	1	1	
1	1	0	0	0	
0	0	0	0	0	
1	1	0	0	0	
1	2	1	0	0	
0	0	0	0	1	
0	0	0	0	1	
0	0	0	0	1	
0	0	0	0	1	
1	1	0	0	0	
1	1	0	0	0	
1	2	1	0	0	
1	1	0	0	1	
1	2	0	1	1	
0	0	0	0	0	
1	1	0	0	0	
1	1	0	0	0	
1	1	0	0	0	
1	1	0	0	0	
1	2	1	0	0	
0	0	0	0	1	
1	2	1	0	1	
1	1	0	0	0	
1	2	1	0	0	
1	2	1	0	0	
1	2	1	0	1	
0	0	0	0	0	
1	1	0	0	1	
1	1	0	0	0	
1	1	0	0	0	
1	1	0	0	0	
1	2	0	0	1	
0	0	0	0	0	

1	1	0	0	0
1	1	0	0	0
0	0	0	0	0
1	2	1	0	0
0	0	0	0	1
0	0	0	0	0
1	1	0	0	0
1	1	0	0	1
1	2	1	1	1
1	1	0	0	1
1	1	0	0	1
1	1	0	0	1
1	1	0	0	0
1	1	0	0	0
1	1	0	0	0
1	2	1	0	0
1	1	0	0	1
0	0	0	0	0
1	1	0	0	0
1	1	0	0	0
1	2	1	1	1
1	1	0	0	0
1	2	1	0	0
0	0	0	0	0
1	2	0	0	1
1	1	0	0	0
1	1	0	0	0
1	1	0	0	0
1	1	0	0	1
1	2	1	0	1
1	1	0	0	1
1	1	0	0	0
0	0	0	0	1
0	0	0	0	0
1	1	0	0	0
1	2	1	0	1
1	1	0	0	0
1	1	0	0	1
1	2	0	1	1
1	1	0	0	0
1	2	1	0	1
1	1	0	0	0
0	0	0	0	1
0	0	0	0	0
1	1	0	0	0

1	2	1	0	0
1	2	0	0	1
1	1	0	0	1
0	0	0	0	0
1	2	0	0	1
1	1	0	0	1
1	1	0	0	0
0	0	0	0	1
1	2	1	0	0
0	0	0	0	0
1	1	0	0	0
1	1	0	0	0
1	2	1	0	1
0	0	0	0	0
1	1	0	0	1
0	0	0	0	0
0	0	0	0	0
1	2	1	0	0
0	0	0	0	1
0	0	0	0	0
1	1	0	0	0
1	2	1	1	1
1	2	1	0	0
0	0	0	0	1
1	2	1	0	1
1	2	1	1	1
0	0	0	0	0
1	1	0	0	0
1	1	0	0	0
0	0	0	0	0
1	1	0	0	0
0	0	0	0	0
1	1	0	0	0
0	0	0	0	0
0	0	0	0	0
0	0	0	0	1
0	0	0	0	0
1	2	1	0	0
0	0	0	0	0
0	0	0	0	0
1	2	1	0	1
1	1	0	0	0
1	1	0	0	0
1	1	0	0	1
0	0	0	0	0

0	0	0	0	1
0	0	0	0	0
0	0	0	0	0
0	0	0	0	1
1	1	0	0	0
0	0	0	0	0
0	0	0	0	0
1	1	0	0	0
1	2	1	0	0
1	2	1	0	0
1	2	0	1	1
0	0	0	0	0
0	0	0	0	1
1	2	1	0	1
0	0	0	0	0
0	0	0	0	0
1	1	0	0	0
1	1	0	0	0
0	0	0	0	1
1	1	0	0	0
1	2	1	0	0
0	0	0	0	0
1	1	0	0	1
1	1	0	0	0
0	0	0	0	0
0	0	0	0	0
0	0	0	0	0
0	0	0	0	0
1	1	0	0	1
1	1	0	0	1
1	1	0	0	0
0	0	0	0	0
0	0	0	0	0
1	2	1	0	1
1	1	0	0	0
0	0	0	0	0
1	1	0	0	0
0	0	0	0	1
1	2	1	0	1
1	1	0	0	0
0	0	0	0	1
1	2	1	0	0
1	1	0	0	1
1	1	0	0	1
1	1	0	0	0

0	0	0	0	1
0	0	0	0	1
0	0	0	0	0
1	1	0	0	0
0	0	0	0	0
0	0	0	0	1
0	0	0	0	1
0	0	0	0	0
1	1	0	0	0
0	0	0	0	1
0	0	0	0	0
1	2	1	0	0
0	0	0	0	0
1	2	1	0	1
0	0	0	0	0
1	1	0	0	0
0	0	0	0	0
0	0	0	0	0
0	0	0	0	1
0	0	0	0	1
1	1	0	0	1
0	0	0	0	0
1	1	0	0	0
0	0	0	0	0
0	0	0	0	1
0	0	0	0	1
0	0	0	0	0
0	0	0	0	0
0	0	0	0	0
0	0	0	0	0
0	0	0	0	0
0	0	0	0	0
1	2	1	0	0
0	0	0	0	0
1	2	1	0	0
1	2	0	1	1
1	2	1	1	1
1	2	1	0	1
1	1	0	0	0
0	0	0	0	0
1	2	0	0	1
0	0	0	0	0
1	2	0	1	1
0	0	0	0	0
0	0	0	0	0

0	0	0	0	1
1	1	0	0	0
1	1	0	0	0
1	1	0	0	0
0	0	0	0	0
1	1	0	0	1
1	2	1	0	0
0	0	0	0	1
0	0	0	0	0
0	0	0	0	0
1	2	1	0	0
0	0	0	0	1
0	0	0	0	0
1	2	1	0	0
0	0	0	0	1
1	2	0	1	1
0	0	0	0	0
0	0	0	0	0
0	0	0	0	0
1	2	1	0	0
1	2	1	0	0
1	1	0	0	0
1	2	0	1	1
0	0	0	0	0
0	0	0	0	0
0	0	0	0	0
1	1	0	0	0
0	0	0	0	0
1	1	0	0	0
0	0	0	0	0
1	1	0	0	1
1	1	0	0	0
1	2	1	0	0
1	1	0	0	0
0	0	0	0	0
0	0	0	0	0
1	1	0	0	0
1	1	0	0	0
0	0	0	0	0
1	2	1	0	0
1	1	0	0	1
1	1	0	0	0
0	0	0	0	1
0	0	0	0	0
1	1	0	0	1

1	1	0	0	1
0	0	0	0	0
0	0	0	0	0
1	1	0	0	1
0	0	0	0	0
0	0	0	0	0
1	1	0	0	0
1	1	0	0	0
1	2	1	0	1
0	0	0	0	0
1	2	1	0	0
1	1	0	0	1
0	0	0	0	1
0	0	0	0	0
0	0	0	0	1
1	2	0	1	1
1	2	1	0	0
1	2	1	0	0
0	0	0	0	0
1	1	0	0	1
0	0	0	0	0
1	1	0	0	0
0	0	0	0	1
1	1	0	0	0
1	1	0	0	1
0	0	0	0	0
0	0	0	0	0
0	0	0	0	0
1	1	0	0	0
0	0	0	0	0
1	1	0	0	0
1	1	0	0	0
0	0	0	0	0
1	2	1	0	0
0	0	0	0	1
1	2	1	0	0
1	2	1	0	1
0	0	0	0	0
1	1	0	0	1
0	0	0	0	1
0	0	0	0	0
0	0	0	0	0
0	0	0	0	0
1	1	0	0	0
1	2	1	0	0

1	2	1	1	1
1	2	1	0	0
1	1	0	0	0
0	0	0	0	0
1	1	0	0	0
0	0	0	0	0
1	2	1	0	0
0	0	0	0	0
0	0	0	0	0
0	0	0	0	0
1	1	0	0	1
1	1	0	0	0
0	0	0	0	0
0	0	0	0	1
0	0	0	0	1
1	2	1	1	1
1	1	0	0	0
1	1	0	0	1
1	2	1	0	0
1	1	0	0	0
0	0	0	0	1
1	1	0	0	1
0	0	0	0	0
1	1	0	0	0
0	0	0	0	0
0	0	0	0	0
1	1	0	0	0
1	2	1	0	1
0	0	0	0	1
1	2	1	0	0
0	0	0	0	0
0	0	0	0	0
1	1	0	0	0
1	2	1	0	0
0	0	0	0	0
0	0	0	0	1
1	2	1	1	1
1	1	0	0	0
1	1	0	0	0
0	0	0	0	1
1	1	0	0	0
1	2	1	0	1
0	0	0	0	0
1	1	0	0	0
1	1	0	0	0

1	2	1	0	0
1	1	0	0	0
1	1	0	0	0
1	2	1	0	0
1	2	1	0	0
0	0	0	0	0
1	1	0	0	0
1	1	0	0	0
1	1	0	0	1
0	0	0	0	0
1	1	0	0	0
1	2	1	0	0
1	2	0	1	1
1	2	0	1	1
0	0	0	0	0
0	0	0	0	0
1	1	0	0	0
0	0	0	0	0
1	2	1	0	0
0	0	0	0	1
0	0	0	0	1
0	0	0	0	1
0	0	0	0	1
1	1	0	0	1
1	1	0	0	0
0	0	0	0	0
0	0	0	0	0
1	1	0	0	0

border or high triglyd	border or highLDL	border or low HDL	border or high non-HDL chol	obesity	nutrition 2
0	0	1	0	0	0
1	0	0	1	0	0
0	0	1	0	0	0
0	1	0	1	0	0
1	0	0	1	0	0
0	0	1	0	0	0
1	0	1	0	0	0
1	0	1	0	0	0
0	1	1	1	0	0
1	0	0	0	0	0
1	1	1	1	0	0
0	0	1	0	0	0
0	0	0	0	0	0
1	0	1	1	0	0
1	0	1	0	0	0
0	1	0	1	0	1
0	1	1	1	0	0
1	1	1	1	0	0
1	1	0	1	0	0
0	0	1	0	0	0
1	0	1	0	0	0
1	0	1	0	0	0
0	1	0	1	0	0
1	1	1	1	0	0
0	0	0	0	0	0
0	0	1	0	1	2
1	0	1	0	0	0
1	0	1	0	0	0
1	0	1	0	0	0
1	0	1	0	0	0
1	0	0	1	0	0
1	1	1	1	0	0
0	0	1	0	1	2
1	0	1	0	0	0
1	0	1	0	0	0
1	1	1	1	0	0
0	1	1	1	0	0
1	1	1	1	0	0
1	0	1	0	0	0
1	0	1	0	0	0
1	0	1	0	0	0
1	1	1	1	0	0
0	0	0	0	0	0

0	1	1	1	0	0
0	0	1	0	0	1
0	0	1	0	0	0
1	0	1	1	0	0
0	1	0	1	0	0
1	0	1	0	0	1
0	0	1	0	0	0
1	1	1	1	0	1
1	1	1	1	0	0
1	1	0	1	0	0
1	1	0	1	0	0
0	1	1	1	0	0
0	1	1	1	0	0
0	1	1	1	0	0
1	1	1	1	0	0
1	0	1	0	0	0
1	1	1	1	0	0
0	0	0	0	0	0
0	0	1	0	0	0
0	0	1	0	0	0
1	1	1	1	0	0
0	0	1	0	0	0
1	0	1	0	0	0
0	0	1	0	0	0
0	1	1	1	0	0
0	0	1	0	0	0
1	0	1	0	0	0
1	0	0	0	0	0
1	1	1	1	0	0
1	1	1	1	0	0
1	0	1	1	0	0
1	0	1	0	0	0
0	1	0	1	0	0
0	0	0	0	0	0
0	0	1	0	0	0
1	1	1	1	1	2
0	0	1	0	1	2
0	1	0	1	0	1
1	1	0	1	0	0
0	0	1	0	0	0
1	1	1	1	0	0
1	0	1	0	0	0
0	1	0	1	0	0
1	0	0	0	0	0
1	0	1	0	0	1

1	0	1	0	0	1
0	1	1	1	0	0
1	1	1	1	0	0
0	0	0	0	0	0
1	1	1	1	0	1
1	1	1	1	1	2
0	0	1	0	0	0
0	1	0	1	0	0
1	0	1	0	0	0
1	0	0	0	0	0
1	0	1	0	0	0
0	1	1	1	0	0
1	1	1	1	0	0
0	0	0	0	1	2
1	1	1	1	0	1
0	1	1	1	0	0
0	0	0	0	0	0
1	0	1	0	0	0
0	1	0	1	0	1
1	0	0	0	0	0
1	0	1	0	0	0
1	1	1	1	1	2
1	0	1	0	0	0
0	0	0	0	1	2
1	1	1	1	0	0
1	1	1	1	0	0
0	0	1	0	0	0
0	1	1	1	0	0
0	0	1	0	0	0
1	0	1	0	0	0
1	1	1	1	0	0
0	0	1	0	0	0
0	0	1	0	0	0
0	0	0	0	0	0
0	0	1	0	0	0
1	1	0	1	0	1
0	0	0	0	0	0
1	0	1	0	0	0
1	0	0	0	0	0
0	0	0	0	0	0
1	1	1	1	0	0
0	0	1	0	0	1
1	0	1	0	0	0
0	1	0	1	0	0
1	0	1	0	0	0

1	1	0	1	0	0
1	0	1	1	0	0
1	0	0	1	0	0
0	1	0	1	0	0
0	0	1	0	0	0
0	0	0	0	0	0
0	0	0	0	0	0
0	0	1	0	1	2
1	1	1	1	0	0
1	0	1	1	0	0
1	1	0	1	0	0
0	0	0	0	0	0
1	0	0	0	0	0
1	0	1	1	0	1
0	1	1	0	0	0
0	0	1	0	0	0
0	0	1	0	0	0
0	0	1	0	0	0
1	1	1	1	0	0
0	0	1	0	0	0
1	0	1	0	0	0
0	0	1	0	0	0
0	1	1	1	0	0
1	0	1	0	0	0
0	0	0	0	0	1
0	0	0	0	0	0
0	0	1	0	0	0
0	0	0	0	0	1
1	0	0	0	1	2
0	1	0	1	0	0
0	0	1	0	0	0
1	0	0	0	0	0
1	0	1	0	0	0
1	1	1	1	0	0
0	0	1	0	0	0
0	0	0	0	0	0
1	0	0	0	0	0
0	0	0	0	0	0
1	1	1	1	0	0
1	0	1	1	0	0
1	0	0	1	0	0
1	0	1	0	0	0
0	1	0	1	0	0
1	1	0	1	0	0
0	0	1	0	0	0

0	0	0	1	0	0
0	0	0	0	0	0
0	0	0	0	0	0
0	0	1	0	0	1
0	0	0	0	0	0
1	0	0	1	0	1
1	0	0	0	0	1
0	0	0	0	0	0
0	0	1	0	0	0
0	1	0	1	0	1
0	0	0	0	0	0
1	0	1	1	0	0
0	0	1	0	0	1
1	0	1	1	0	0
0	0	0	0	0	0
0	0	1	0	0	0
0	0	0	0	0	0
0	0	1	0	0	0
0	0	0	0	0	0
0	1	0	1	0	0
0	1	1	1	0	0
1	0	1	0	0	0
0	1	1	1	1	2
0	0	0	0	0	0
0	0	0	0	0	0
1	0	0	0	0	0
0	0	0	0	0	0
1	0	0	0	0	0
1	0	0	0	0	1
0	0	0	0	0	0
0	0	0	0	0	1
1	0	0	0	0	0
1	0	1	1	0	0
0	0	0	0	0	0
1	0	1	0	0	0
1	1	0	1	0	0
1	1	1	1	0	0
1	1	1	1	0	0
0	0	1	0	0	0
1	0	1	0	0	0
1	1	1	1	0	1
0	0	0	0	0	0
1	1	0	1	0	0
1	0	0	0	0	0
0	0	1	0	0	0

0	1	1	1	0	0
0	0	1	0	0	0
1	0	1	0	0	0
1	0	1	0	0	0
1	0	1	0	0	0
1	1	1	1	0	0
1	0	1	1	0	0
1	1	1	1	0	0
1	0	0	0	0	0
1	0	1	0	0	0
1	0	1	0	0	0
1	1	1	1	0	0
1	0	0	0	0	0
1	0	1	1	0	1
0	0	0	0	0	0
1	1	0	1	0	0
0	0	0	0	0	0
0	0	0	0	0	0
0	0	1	0	0	0
1	0	1	0	0	0
1	0	1	0	0	0
1	0	1	0	0	0
1	1	1	1	0	0
0	0	0	0	0	0
0	0	0	0	0	1
1	0	0	0	0	0
1	0	0	0	0	0
1	0	1	0	0	0
0	0	1	0	0	0
0	0	0	0	0	0
1	1	1	1	0	1
0	0	1	0	1	2
1	0	1	0	0	0
1	0	1	0	0	0
0	0	1	0	0	0
0	0	1	0	0	0
0	0	1	0	0	0
0	0	1	0	0	0
1	0	1	0	0	0
1	0	1	0	0	0
1	0	1	1	0	0
0	0	1	0	0	0
0	0	0	0	0	0
1	0	1	0	0	0
1	0	1	1	0	0

1	0	0	0	1	2
1	0	0	0	0	0
1	0	0	0	0	0
1	1	1	1	0	0
1	0	1	0	0	1
0	0	1	1	0	0
0	0	1	0	0	0
0	0	1	0	0	0
1	1	1	1	1	2
1	0	1	0	0	1
1	0	1	1	0	0
0	1	1	1	0	0
0	1	1	1	0	0
0	0	0	0	0	0
1	0	1	1	0	0
1	1	0	1	0	1
1	0	1	0	0	0
1	0	1	1	0	1
0	1	1	1	1	2
1	1	1	1	0	1
0	0	1	0	0	0
1	0	1	0	0	0
0	1	0	1	0	0
0	0	1	0	0	0
1	1	0	1	0	0
0	0	1	0	0	0
0	0	0	0	0	0
0	0	1	0	0	0
0	0	1	1	0	0
0	0	1	0	0	0
0	0	1	0	0	0
1	0	1	0	0	0
1	0	0	0	0	0
1	0	1	1	0	0
0	1	0	1	0	0
1	0	1	1	0	1
1	1	1	1	0	0
0	0	1	0	0	0
1	0	1	1	0	0
1	1	1	1	0	0
0	0	0	0	0	0
0	0	1	0	0	0
0	0	1	1	0	0
1	0	1	0	0	0
1	0	1	1	0	0

1	1	1	1	0	0
1	0	1	0	0	1
0	0	1	0	0	0
1	0	1	0	0	0
0	0	1	0	0	0
0	0	0	0	0	0
1	0	1	0	0	0
0	0	0	0	0	0
1	0	0	0	0	0
1	0	1	0	0	0
1	0	1	1	0	0
1	0	1	1	0	0
1	0	1	0	0	0
1	0	1	1	0	0
1	0	1	1	0	0
1	1	1	1	0	0
0	0	1	0	0	0
1	1	1	1	0	0
1	0	1	0	1	2
0	0	1	0	0	1
0	1	0	1	0	0
1	1	1	1	0	0
0	0	0	0	0	1
1	0	1	0	0	0
0	0	1	0	0	0
1	0	1	0	0	0
1	0	1	0	0	0
1	0	1	1	0	0
1	0	0	0	0	0
1	0	1	0	0	1
1	0	1	0	0	0
0	0	1	0	0	0
0	0	1	0	0	0
1	0	1	0	0	0
1	0	0	0	0	0
1	1	1	1	0	0
1	1	1	1	0	0
0	0	1	0	0	1
1	0	1	0	0	0
1	1	0	1	0	0
1	0	1	1	0	0
1	1	1	1	0	1
1	0	0	0	0	0
1	0	1	0	0	0
1	0	1	1	1	2

1	0	1	1	0	0
1	0	1	0	0	1
1	0	1	0	0	0
1	0	1	1	0	0
1	0	1	0	0	0
0	0	0	0	0	0
1	0	1	0	0	0
1	0	1	1	0	0
1	1	1	1	0	0
1	0	0	0	0	0
1	0	1	0	0	0
1	0	1	0	0	0
1	1	0	1	0	0
1	1	0	1	0	0
0	0	1	0	0	0
0	0	0	0	0	0
0	1	1	1	0	0
0	0	0	0	0	0
1	0	1	0	0	0
0	0	0	0	0	1
0	1	1	1	0	0
0	1	0	1	0	0
0	1	1	1	0	0
1	1	0	1	1	2
0	0	1	0	0	0
0	0	1	1	0	0
1	0	1	1	0	0
1	0	1	0	0	1
